# Is Transthyretin a Regulator of Ubc9 SUMOylation?

**DOI:** 10.1371/journal.pone.0160536

**Published:** 2016-08-08

**Authors:** Elżbieta Wieczorek, Sylwia Kędracka–Krok, Katarzyna Sołtys, Urszula Jankowska, Rafał Hołubowicz, Justyna Seliga, Andrzej Ożyhar

**Affiliations:** 1 Department of Biochemistry, Faculty of Chemistry, Wrocław University of Science and Technology, Wybrzeże Wyspiańskiego 27, Wrocław, Poland; 2 Department of Physical Biochemistry, Faculty of Biochemistry, Biophysics and Biotechnology, Jagiellonian University, Krakow, Poland; 3 Department of Structural Biology, Malopolska Centre of Biotechnology, Jagiellonian University, Krakow, Poland; Istituto di Genetica Molecolare, ITALY

## Abstract

Ageing and mutations of transthyretin (TTR), the thyroid hormones and retinol transporting protein lead to amyloidosis by destabilizing the structure of TTR. Because protein structure is regulated through posttranslational modifications, we investigated the Small Ubiquitin-like Modifier (SUMO)ylation of TTR. We chose the widely used Ubc9 fusion-directed SUMOylation system, which is based on a fusion of the SUMOylation substrate of interest with Ubc9, a sole SUMO conjugating enzyme. Surprisingly, despite our presumptions, we found that Ubc9 fused to TTR was SUMOylated at a unique set of lysine residues. Three unknown SUMOylation sites of Ubc9—K154, K18 and K65—were revealed by mass spectrometry (MS). The previously reported SUMOylation at K49 of Ubc9 was also observed. SUMOylation of the lysine residues of TTR fused to Ubc9 was hardly detectable. However, non-fused TTR was SUMOylated *via* trans-SUMOylation by Ubc9 fused to TTR. Interestingly, mutating the catalytic residue of Ubc9 fused to TTR did not result in complete loss of the SUMOylation signal, suggesting that Ubc9 linked to TTR is directly cross-SUMOylated by the SUMO-activating enzyme E1. Ubc9, TTR or fusion proteins composed of TTR and Ubc9 specifically affected the global SUMOylation of cellular proteins. TTR or Ubc9 alone increased global SUMOylation, whereas concomitant presence of TTR and Ubc9 did not further increase the amount of high-molecular weight (HMW) SUMO conjugates. Our data suggest that TTR may influence the SUMOylation of Ubc9, thereby altering signalling pathways in the cell.

## Introduction

Transthyretin (TTR) is an all-beta 55-kDa homo-tetrameric protein [1,2], which binds thyroid hormones and distributes retinol indirectly *via* complexing retinol binding protein [2,3]. The major sources of TTR are the liver and the brain, which supply the serum and cerebrospinal fluid pools, respectively [4,5]. An increasing number of reports, however, document the presence of TTR in many other types of cells, including those that apparently do not synthesize TTR [4,6–9]. TTR mRNA or TTR protein have been found in retinal pigmented epithelial cells [10], leukocytes [9], pancreatic islets [11], dorsal root ganglia [12], ependymoma cells [7] and others [4]. Circulating TTR can also be internalized [7]. A number of reports suggest that TTR is not a simple hormone-transporting protein. Cryptic proteolytic activity of TTR has been observed [13], and TTR has been found to participate in diverse intracellular processes, such as the cleavage of neuropeptide Y [14,15], the modulation of behaviour [16,17], cognition [18], the regulation of the 14-3-3 protein level and autophagy [19]. TTR also participates in nerve physiology, accelerates nerve regeneration [12,17] and exhibits protective effects in Alzheimer’s disease [20,21]. Recently, TTR expression has been shown to be regulated by heat shock factor 1 (HSF1) in neuronal cells in response to stress [22]. Outside of the nervous system, TTR plays an important role in stimulus-secretion coupling in β-cells [23] and glucose homeostasis in α-cells of the pancreas [24]. Importantly, the misfolding of wild-type TTR results in senile cardiac amyloidosis [25]. Moreover, most of the more than 100 mutations of TTR are associated with pathological phenotypes of familial amyloidotic polyneuropathy [26,27]. These observations suggest that the intact, higher-order structure of TTR is a prerequisite for protection against disease [25,27,28]. In protein-misfolding disorders, one of the key factors protecting against pathogenesis is the proper functioning of the ubiquitin-dependent proteasomal system “cross-talking” with other post-translational modifications (PTMs) [29]. Proteins that form amyloid deposits are extensively post-translationally modified [30–32]. Modification of the TTR structure also strongly affects the process of fibril formation [2,27,28]. Small Ubiquitin-like Modifier (SUMO)ylation is among the modifications that influence protein structure and function [33–36]; therefore, we decided to verify whether TTR is modified by SUMO-conjugation. Although none of the eight lysine residues of human TTR possess the consensus SUMOylation motif (ΦKXE, where Φis aromatic, and X is any amino acid) [37], several reports have noted that SUMOylation also occurs at non-consensus sites [33,34,38].

SUMOylation is a highly dynamic and spatially defined process that regulates multiple cellular events [33] and requires the coordinated activity of the SUMOylation machinery: activating enzyme (E1), conjugating enzyme (E2), SUMO ligases (E3) and deSUMOylating isopeptidases cooperating with SUMO-targeted ubiquitin ligases [33,34]. SUMOylation is difficult to observe because of its dynamic nature. In addition, only a very small portion of the total pool of the substrate protein is typically SUMOylated at any particular moment [33,34,39,40]. Therefore, we decided to investigate the SUMOylation of TTR in a system that facilitates contact between the sole SUMO-conjugating enzyme (Ubc9) and the substrate protein. This system is called Ubc9 fusion-directed SUMOylation (UFDS) because the protein of interest is covalently linked to Ubc9 [41]. We observed that the fusion of TTR with Ubc9 resulted in extensive SUMOylation. The modification occurred for the SUMO 1 or SUMO 2/3 isoform and depended on the relative orientation of TTR and Ubc9. Mutation of the lysine residues of Ubc9 and TTR revealed that most of the SUMO-conjugation occurred on the Ubc9 moiety. Mass spectrometry (MS) showed that K154 of Ubc9 was the primary SUMOylation site and that K18, K49 and K65 of Ubc9 were also SUMOylated. SUMOylation of the TTR portion was minor and most likely occurred at K126. However, TTR that was not fused to Ubc9 could be trans-SUMOylated more efficiently. Surprisingly, fusion proteins composed of TTR and the catalytic site mutant of Ubc9 were also SUMOylated, but the formation of SUMO chains was completely abolished. Preliminary experiments indicated that TTR modulates general protein SUMOylation, possibly by influencing SUMO conjugation to Ubc9 lysine residues and, consequently, the substrate specificity of Ubc9. In conclusion, aiming to analyse the SUMOylation of TTR, we determined the SUMOylation of Ubc9 fused to TTR and the regulatory effect of TTR on global SUMOylation of cellular proteins.

## Results

### SUMOylation of TTR in UFDS

**Fig 1:**
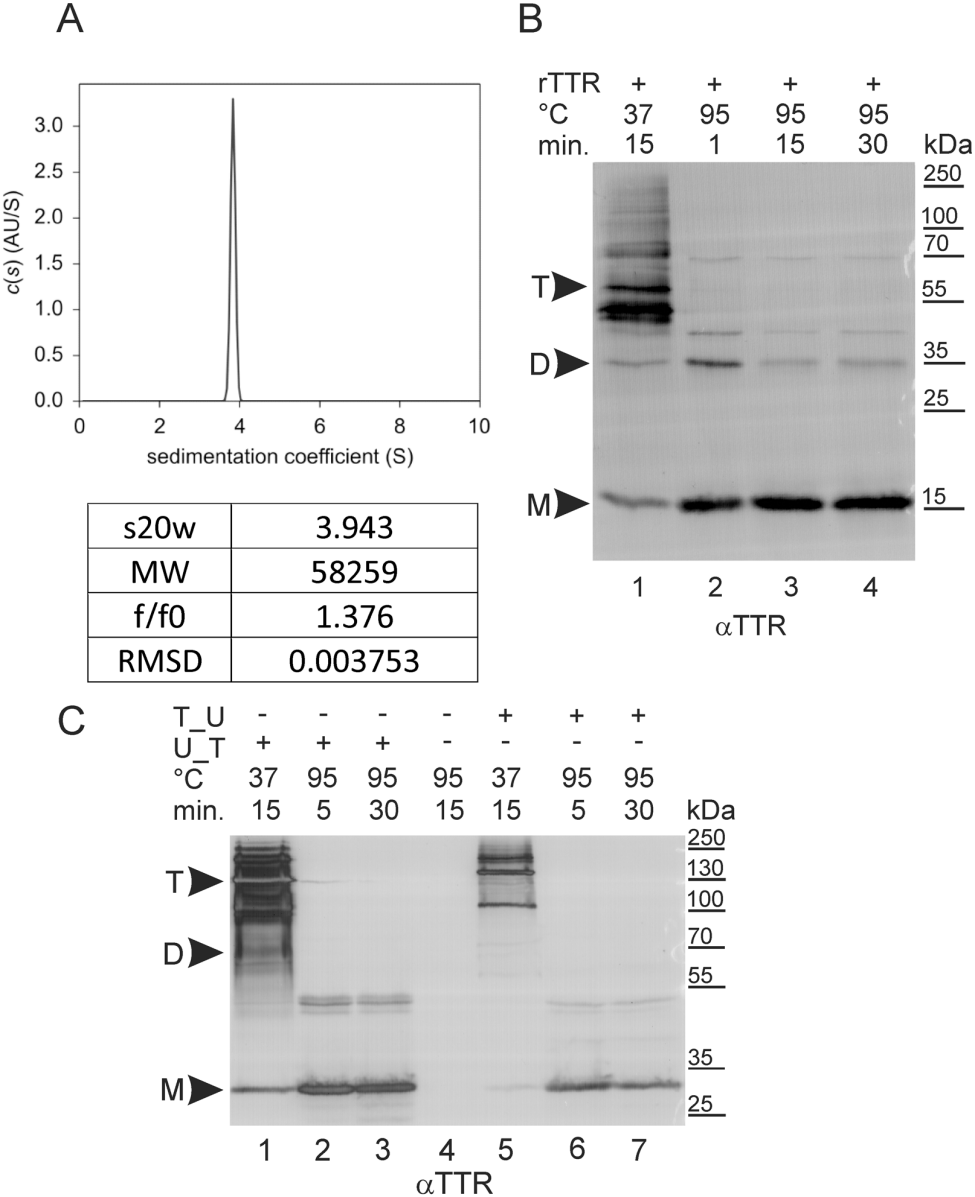
Quaternary structure of TTR_Ubc9, Ubc9_TTR and recombinant non-fused TTR. Recombinant, non-fused TTR (rTTR) was expressed in *Escherichia coli*, purified to homogeneity and subjected to ultracentrifugation (A). TTR fused to the C-terminus (U_T) or N-terminus (T_U) of Ubc9 was expressed in the HEK293 cell line. Purified rTTR samples (B) and HEK293 cell lysates (C) were analysed by WB using anti-TTR antibodies (αTTR). The denaturation time and temperature are indicated at the tops of the blots. M, D and T indicate the expected MWs of the monomers (32 kDa), dimers (64 kDa) and tetramers (128 kDa) of the analysed proteins, respectively.

To study the SUMOylation of TTR, we used a system that enables the detection of SUMOylation products and has been successfully used to study the SUMOylation of other proteins [42,43]. The protein of interest (in this case, TTR) is fused to Ubc9 and co-expressed with exogenous enhanced green fluorescent protein (EGFP)-labelled SUMO-1 (ES1) or SUMO-3 (ES3) in the human embryonic kidney 293 (HEK293) cell line. SUMOylation is observed as an upshift in Western blotting (WB) after the separation of cell lysate proteins by sodium dodecyl sulfate polyacrylamide gel electrophoresis (SDS-PAGE) [41]. To ensure that the fusion of TTR to Ubc9 does not disturb tetramer formation, we first compared the molecular properties of human recombinant TTR (rTTR) purified from an *E*. *coli* bacterial system (S1 Fig) and TTR fused either to the N-terminus (TTR_Ubc9) or the C-terminus (Ubc9_TTR) of Ubc9 expressed in a HEK293 cell line, henceforth referred to as fusion proteins. When rTTR was subjected to sedimentation velocity, the sedimentation profile revealed the dominant species at 3.9430 S (>99%), which corresponded to a rTTR tetramer of 58.3 kDa, as shown by the fitted molecular weight (MW) (Fig 1A). In addition, rTTR tetramer molecules were also observed as metastable conformers in SDS-PAGE followed by WB, unless the samples were heat-denatured prior to electrophoresis (Fig 1B). Concomitantly, unheated TTR_Ubc9 or Ubc9_TTR migrated with mobilities equivalent to their tetrameric forms (Fig 1C), suggesting that the fusion of TTR with Ubc9 does not lead to tetramer dissociation and allows the retention of these proteins’ proper quaternary structure.

**Fig 2:**
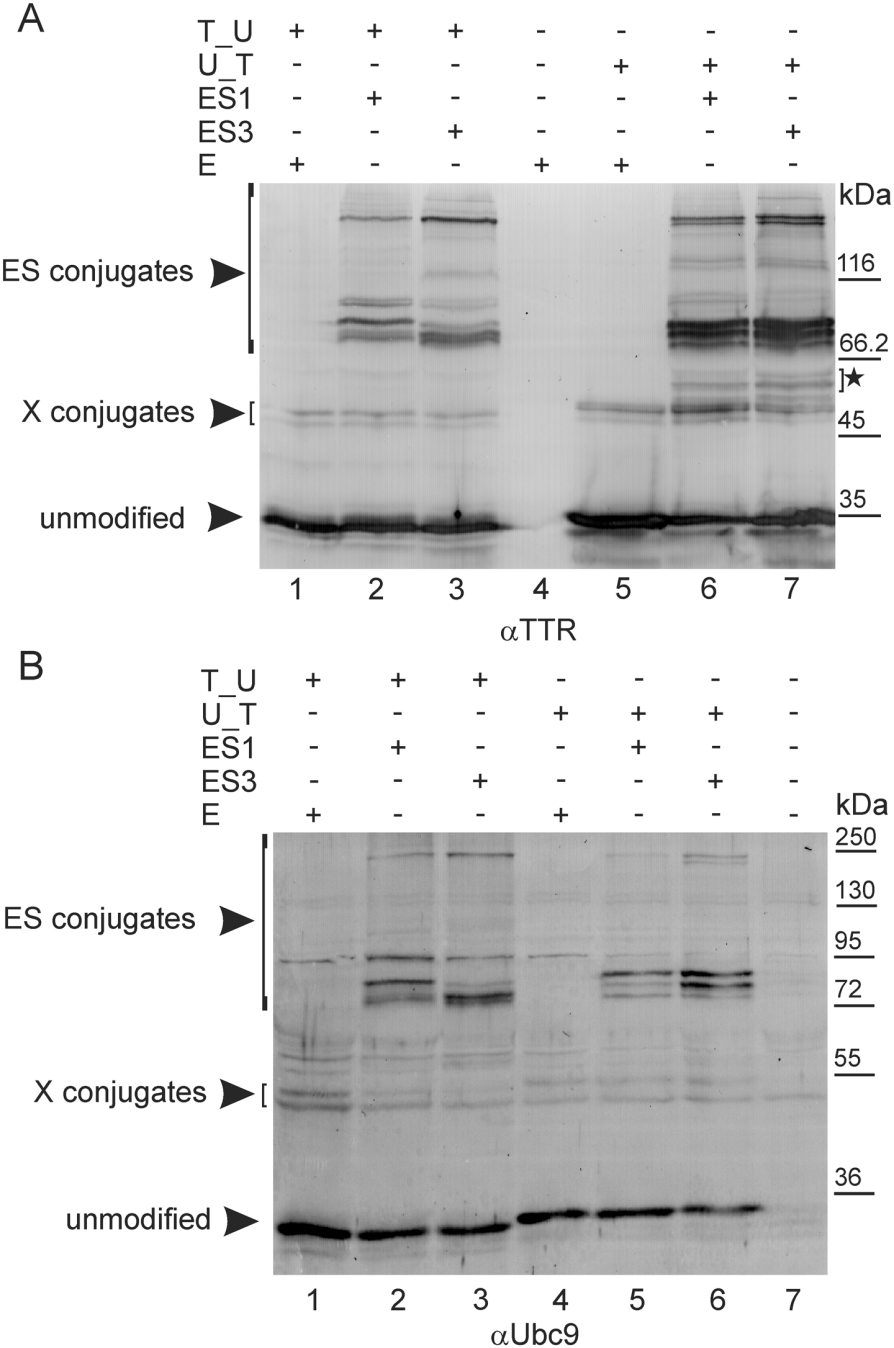
SUMOylation patterns of TTR_Ubc9 and Ubc9_TTR. TTR fused to the N-terminus (T_U) or C-terminus (U_T) of Ubc9 was co-expressed in HEK293 cells with EGFP-labelled SUMO-1 (ES1), SUMO-3 (ES3) or EGFP (E). Cell lysates were analysed by WB using anti-TTR (A) or anti-Ubc9 antibodies (B). The asterisk indicates truncated SUMOylated proteins. ES conjugates and X conjugates indicate forms of T_U and U_T modified by EGFP-labelled SUMO or endogenous modifiers, respectively.

Next, we analysed the SUMOylation of TTR_Ubc9 and Ubc9_TTR using ES1 and ES3 *via* WB analysis with anti-TTR or anti-Ubc9 antibodies (Fig 2). All samples were heat-denatured prior to electrophoresis to ensure the dissociation of tetramers to monomers and enable the unequivocal identification of the SUMOylation products. Unmodified TTR_Ubc9 or Ubc9_TTR monomers, regardless of the arrangement of TTR and Ubc9, migrated in SDS-PAGE slightly below the 35- or 36-kDa marker, which is consistent with the calculated MW of 32 kDa. TTR_Ubc9 always migrated slightly faster than Ubc9_TTR (Fig 2A lanes 1–3 *versus* lanes 5–7 and Fig 2B lanes 1–3 *versus* lanes 4–6), but the smallest polypeptides were more abundant for Ubc9_TTR, suggesting that limited proteolysis might occur. Remarkably, even in the absence of exogenous SUMO, we observed bands with lower mobilities (X conjugates in Fig 2) compared with the unmodified proteins, possibly because of modifications of the fusion proteins by endogenous SUMO or other modifiers of similar MW (11 kDa). The co-expression of ES1 or ES3 with TTR_Ubc9 or Ubc9_TTR resulted in many other specific bands detected with anti-TTR (Fig 2A lanes 2–3 and 6–7) and anti-Ubc9 antibodies (Fig 2B lanes 2–3 and 5–6), forming characteristic and highly reproducible patterns corresponding to ES conjugates. The anti-Ubc9 antibody generated some weak unspecific bands (Fig 2B lane 7), but detection with the anti-TTR antibodies was very specific, and no signal was observed when cell lysates containing EGFP (E) or EGFP-labelled SUMO and Ubc9 were analysed (Fig 2A lane 4 and data not shown). Most of the SUMO conjugates migrated with a mobility corresponding to a MW ranging from 72 to 95 kDa (Fig 2B lanes 2–3 and 5–6 and Fig 2A lanes 2–3 and 6–7), suggesting that one molecule of EGFP-labelled SUMO (40 kDa) is attached to the fusion protein (32 kDa). Interestingly, we observed more than one band in this MW range. The patterns of ES1-conjugation compared to ES3-conjugation appeared to mainly differ in the intensity rather than the presence of a particular band (Fig 2A compare lanes 2 to 3 and lanes 6 to 7). These minor differences suggest that different SUMO isoforms may have different preferences for lysine residues or that the attachment of SUMO to a particular site may be a priming signal for unique combinations with other PTMs, including ubiquitination. Alternatively, high-MW (HMW) bands (above 116 kDa) (Fig 2A and 2B) represent protein conjugates with two or more molecules of EGFP-labelled SUMO. It is important to note that the anti-TTR and anti-Ubc9 polyclonal antibodies produced very similar patterns (compare Fig 2A to 2B), although they both recognized the epitopes, which were differently located in TTR_Ubc9 and Ubc9_TTR. The anti-Ubc9 antibody was raised against the 81-amino acid fragment of the N-terminus of Ubc9, whereas the anti-TTR antibody was raised against full-length TTR. Therefore, we believe that we observed intact SUMOylated forms of TTR_Ubc9 or Ubc9_TTR, except for the bands indicated with an asterisk in Fig 2A. These bands were detected with anti-TTR and not with anti-Ubc9 antibodies only when EGFP-labelled SUMO was co-expressed with fusion proteins. Therefore, these bands apparently represent proteolytic fragments of Ubc9_TTR or, to a lesser extent, TTR_Ubc9, modified by exogenous SUMO. These fragments should contain the TTR portion of the fusion protein, possibly with a small fragment of Ubc9.

In conclusion, the occurrence of many protein forms with different MWs, which were often present in sub-stoichiometric amounts and resulted from the exogenous (or endogenous) modification of fusion proteins by SUMO or other modifiers, suggested that multiple SUMOylation sites exist in TTR_Ubc9 and Ubc9_TTR.

### Site-directed mutagenesis

**Fig 3:**
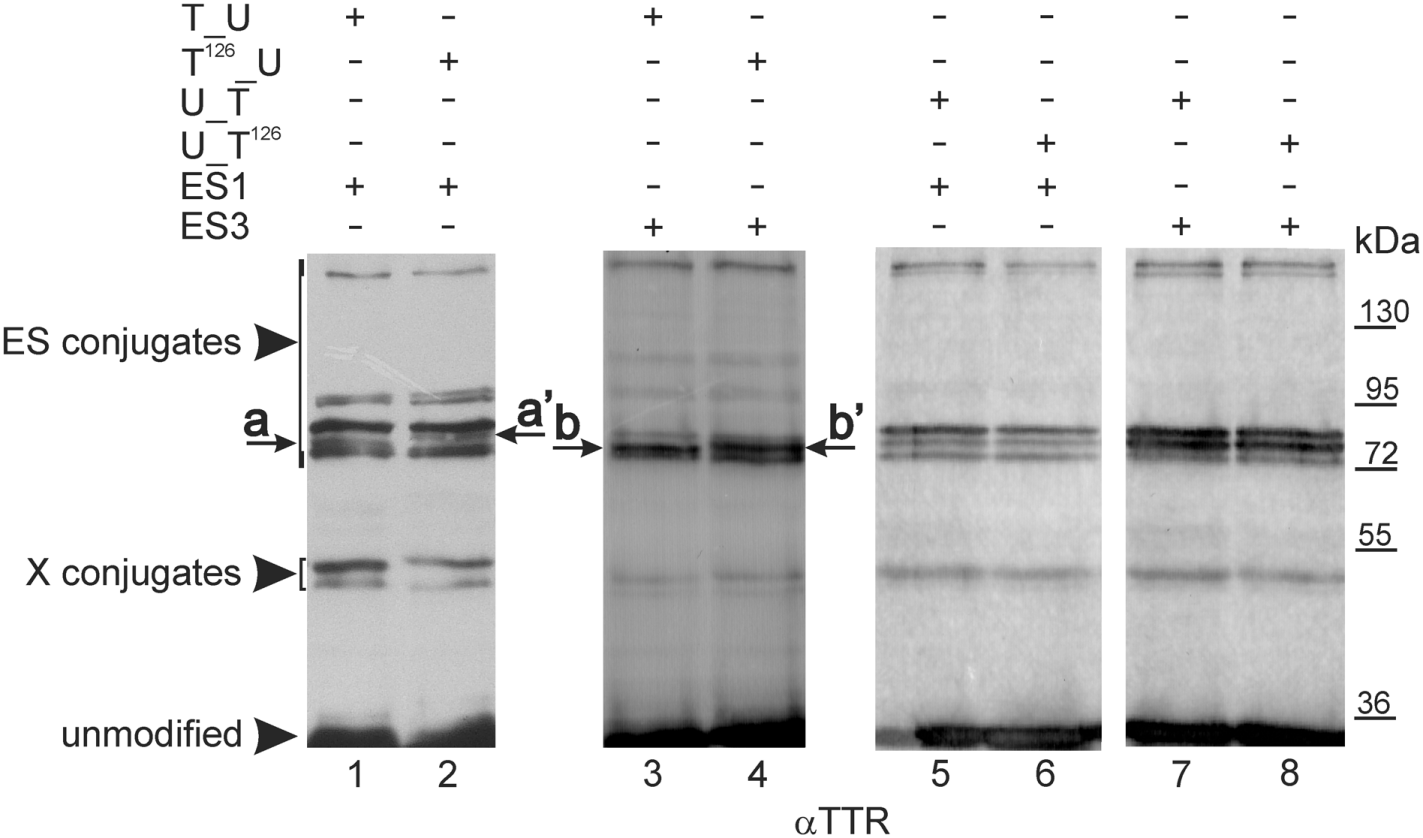
K126R mutation of TTR in TTR_Ubc9 but not in Ubc9_TTR alters the SUMOylation pattern. Non-mutated TTR_Ubc9 (T_U) and Ubc9_TTR (U_T) or the K126R mutant of TTR in TTR^126^_Ubc9 (T^126^_U) or Ubc9_TTR^126^ (U_T^126^) were co-expressed in HEK293 cells with EGFP-labelled SUMO-1 (ES1) or SUMO-3 (ES3). The cell lysates were analysed by WB using anti-TTR antibodies (αTTR). The letters indicate changes in the mobility of the bands resulting from the K126R mutation in TTR. ES conjugates and X conjugates indicate forms of the fusion proteins modified by EGFP-labelled SUMO or endogenous modifiers, respectively.

We used site-directed mutagenesis to determine which lysine residues in the fusion proteins were SUMOylated. We assumed that SUMO is conjugated to TTR, which is the standard assumption of the UFDS system [41]. Initially, we evaluated the potential SUMOylation of eight lysine residues of TTR using bioinformatic tools. However, we obtained contradictory results: PCI-Sumo software predicted that four lysine residues of TTR would be modified by SUMO; Sumo sp 1.0 indicated that only K70 of TTR would be SUMOylated; and SUMOplot found no SUMOylation site in TTR at all (data not shown). Therefore, we obtained the DNA constructs for fusion proteins with point mutations changing the consecutive lysine to arginine residues in TTR and subjected them to UFDS analysis as performed for the non-mutated fusion proteins. A comparison of the SUMOylation patterns obtained for TTR_Ubc9 and TTR^mut^_Ubc9 in experiments conducted with S1E or S3E revealed a difference only for mutant K126R of TTR (TTR^126^_Ubc9) (Fig 3 and S2 Fig). For this mutant, the electrophoretic mobility of one band in the MW range of 72–95 kDa was lower in both the ES1 and ES3 patterns (Fig 3 and S2 Fig, lanes 1–4 bands a, a’ and b, b’). However, the K126R mutant of TTR fused to Ubc9 at the C-terminus (Ubc9_TTR^126^) showed no apparent difference in the intensity or electrophoretic mobility of S1E- or S3E-conjugation patterns compared to Ubc9_TTR (Fig 3, lanes 5–8).

**Fig 4:**
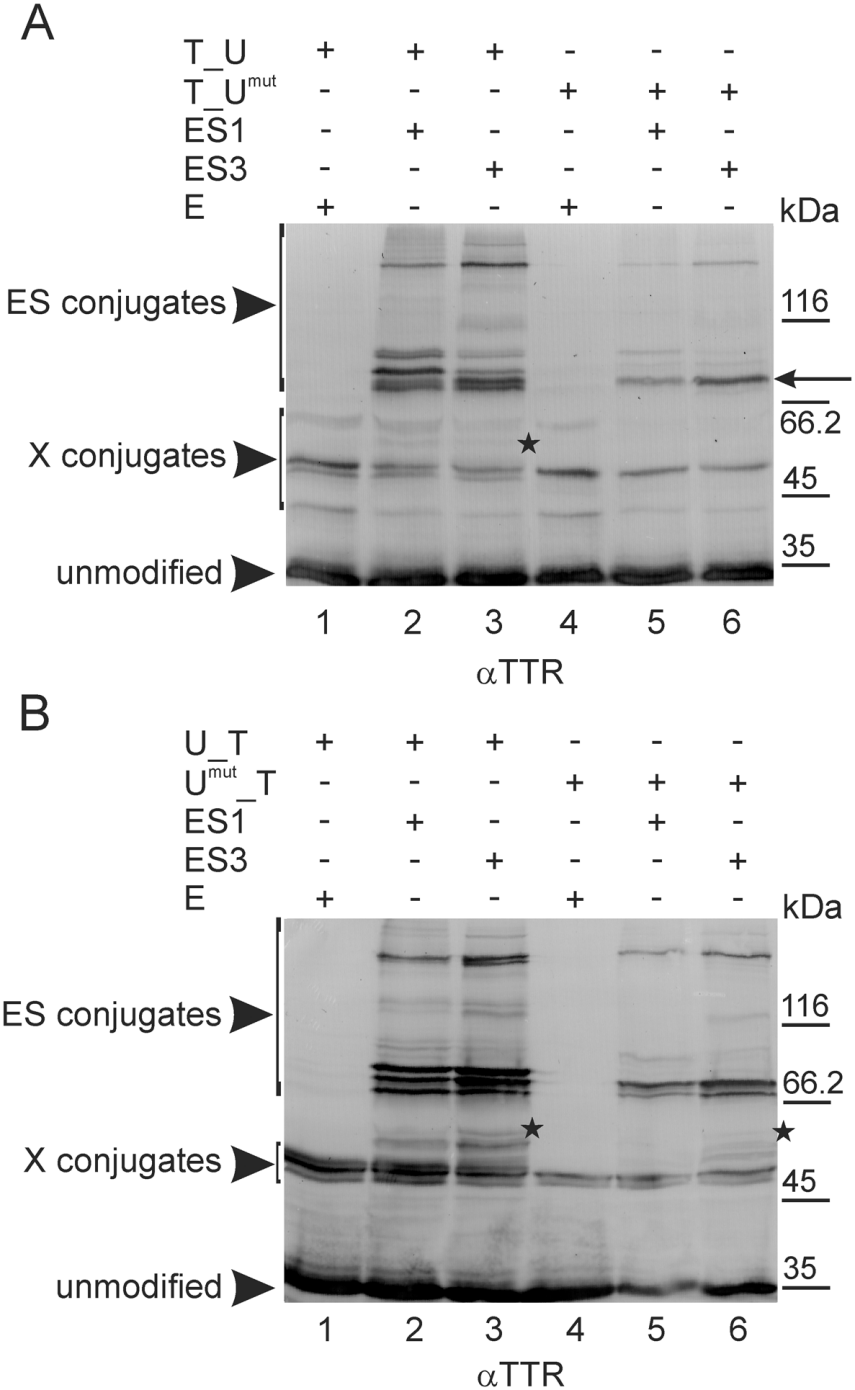
Mutation of the lysine residues of Ubc9 in TTR_Ubc9 and Ubc9_TTR affects the SUMOylation patterns. TTR fused to non-mutated (U) or the K14,153,154R mutant of Ubc9 (U^mut^) located at either the N-terminus (T_U and T_U^mut^) (A) or the C-terminus (U_T and U^mut^_T) (B) was co-expressed with EGFP-labelled SUMO-1 (ES1), SUMO-3 (ES3) or EGFP (E) in the HEK293 cell line. Cell lysates were analysed by WB using anti-TTR antibodies (αTTR). ES conjugates and X conjugates indicate proteins modified by EGFP-labelled SUMO or endogenous modifiers, respectively. The asterisks mark truncated products of SUMOylation. The arrow indicates the band with altered mobility.

The absence of an effect of TTR lysine residue mutations other than K126R on the SUMOylation of TTR^mut^_Ubc9, as well as the lack of an apparent effect of this mutation in the fusion protein with a reversed arrangement of TTR and Ubc9 (Ubc9_TTR^126^) can be explained as follows: (1) K126 of TTR is inaccessible to SUMO-conjugation in Ubc9_TTR; (2) K126R mutation alters the position of the SUMOylation site only in TTR^126^_Ubc9 [40,43]; (3) all analysed fusion proteins with an opposite arrangement of TTR and Ubc9 have different free N-termini, resulting in their distinct localization and, therefore, altering their availability to SUMOylation machinery; and (4) despite our initial assumption, we observed concomitant or exclusive SUMOylation of the lysine residues of Ubc9 in fusion proteins, and the K126R mutation of TTR affects only PTMs of the Ubc9 portion. Some reports have shown that Ubc9 is a substrate for SUMOylation itself and that SUMO molecules are transferred to lysine residues of Ubc9 directly by the activating enzyme E1 (cross-SUMOylation) [33,44,45]. Significant SUMOylation of Ubc9 fused to other proteins, however, has not been generally observed in the UFDS system [41,43]. To ensure that our data, obtained for Ubc9 fused to TTR, are the result of the specific influence of TTR on the SUMOylation of Ubc9, we performed a control experiment. TTR was exchanged with enhanced yellow fluorescent protein (EYFP), and the resulting EYFP_Ubc9 was co-expressed with ES1 or ES3 and analysed using the same methods as for the fusion proteins comprising TTR and Ubc9. Only an insignificant amount of the modified form of EYFP_Ubc9 was observed (data not shown). Therefore, we decided to verify the SUMOylation of fusion proteins composed of TTR and Ubc9 with mutated lysine residues. *In vivo*, K14 of human Ubc9 is a major SUMOylation site [44], but K153 and, later, K49 and K146 have also been shown to be SUMOylated [38,45]. We changed the major (K14), minor (K153) and non-SUMOylated (K154) lysines into arginine residues in Ubc9 and fused this K14,153,154R mutant (Ubc9^mut^) to the N- or C-terminus of TTR. The SUMOylation patterns of TTR_Ubc9^mut^ or Ubc9^mut^_TTR obtained with ES1 or ES3 revealed reduced numbers and intensities of bands compared to the respective non-mutated fusion proteins (Fig 4). In the mobility range relevant to the MW of mono-SUMOylated products (ca. 72–95 kDa, between the 66.2- and 116-kDa markers), one strong- and two moderate-intensity bands were missing for TTR_Ubc9^mut^ (Fig 4A compare lanes 2, 3 to lanes 5, 6), and one strong band was absent for Ubc9^mut^_TTR (Fig 4B compare lanes 2, 3 to lanes 5, 6). Moreover, the mobility of the major remaining band representing the mono-SUMOylated forms of TTR_Ubc9^mut^ (the arrow in Fig 4A lanes 5 and 6) was subtly different from that of all bands observed for TTR_Ubc9 (Fig 4A lanes 2–3), possibly suggesting a change in the site of SUMO attachment. The number of HMW bands was also lower; for example, we observed only one, instead of two, bands of approximately 200 kDa when Ubc9^mut^_TTR was co-expressed with ES1 or ES3 (Fig 4B, compare lanes 2, 3 to lanes 5, 6). The intensity and number of bands representing endogenous modifications (X-conjugation) also decreased (Fig 4A and 4B, compare lanes 1–3 to lanes 4–6). Interestingly, the differences between the ES1 and ES3 patterns of TTR_Ubc9 were reduced for TTR_Ubc9^mut^ (Fig 4A compare lanes 2 to 3 and 5 to 6). The SUMOylation patterns of Ubc9^mut^_TTR obtained for ES1 and ES3 also did not show major differences (Fig 4B). However, the amounts of both the SUMOylated full-length fusion proteins and the proteolytic fragments (indicated by asterisks) were significantly diminished (Fig 4A and 4B compare lanes 2, 3 to lanes 5, 6).

**Fig 5:**
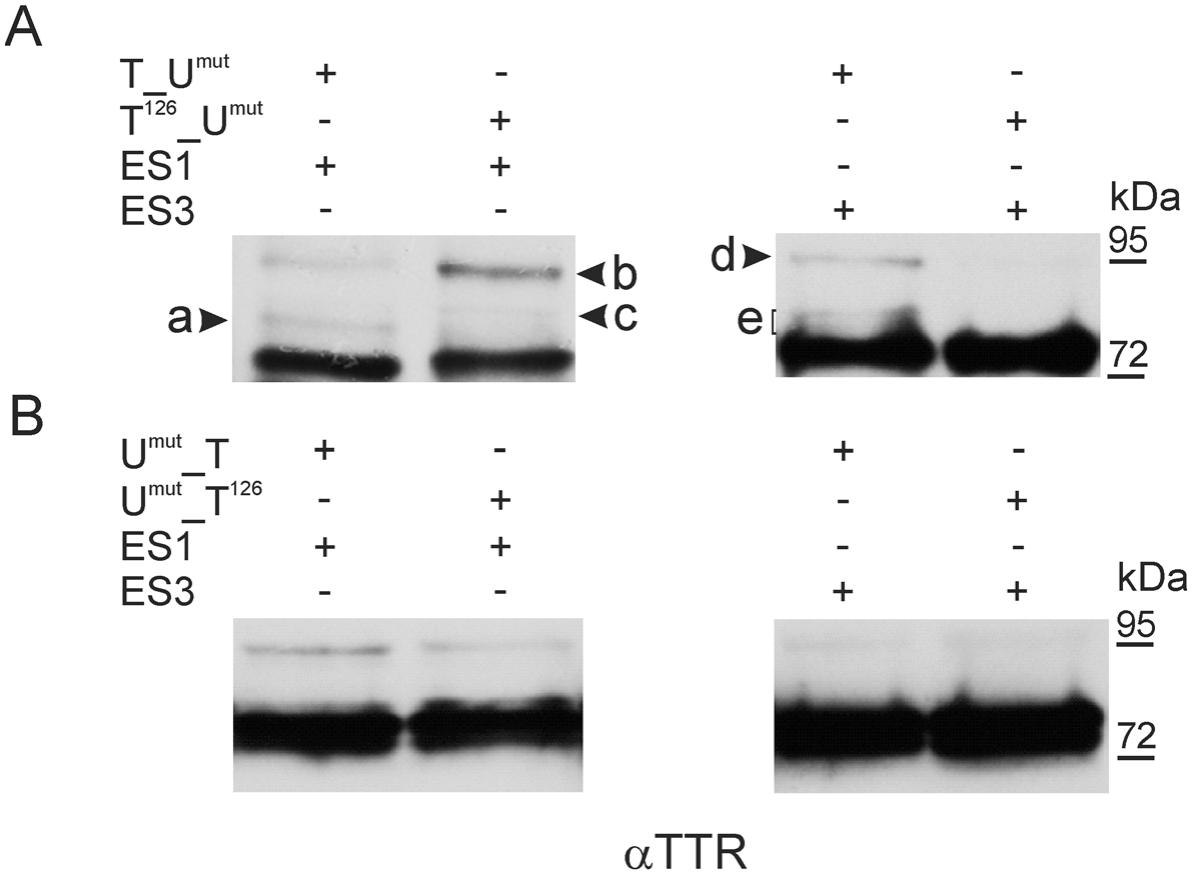
Mutation K126R of TTR alters the SUMOylation patterns of TTR_Ubc9^mut^. Non-mutated TTR (T) or K126R mutant of TTR (T^126^), fused to either the K14,153,154R mutant of Ubc9 (U^mut^) at the N-terminus (T_U^mut^ and T^126^_U^mut^) (A) or the C-terminus (U^mut^_T and U^mut^_T^126^) (B) was co-expressed in HEK293 cells with EGFP-labelled SUMO-1 (ES1) or SUMO-3 (ES3). The cell lysates were analysed by WB using anti-TTR antibodies (αTTR). Arrows with letters (a, b, c, d, or e) indicate band differences caused by the K126R mutation of TTR.

Next, we compared the SUMOylation patterns of consecutive lysine-to-arginine point mutants of TTR (TTR^mut^) fused to the K14,153,154R mutant of Ubc9 (Ubc9^mut^) to those obtained for wild-type TTR fused to Ubc9^mut^. Again, a difference was observed only for TTR^126^_Ubc9^mut^ in the MW range corresponding to the mobility of mono-SUMOylated products (72–95 kDa) (Fig 5A and data not shown). When SUMOylation was probed with ES1, the intensities of two bands increased (bands b and c in Fig 5A), and one band was absent (band a in Fig 5A). SUMOylation of TTR^126^_Ubc9^mut^ with ES3 resulted in the absence of weak double band (band e in Fig 5A), and the intensity of the remaining band (band d in Fig 5A) was significantly lower compared to the SUMOylation pattern of TTR_Ubc9^mut^ (Fig 5A). Note that all bands representing the SUMOylated molecules of TTR_Ubc9^mut^ changed because of the K126R mutation in TTR were considerably fainter than other bands. In contrast, Ubc9^mut^_TTR^126^ did not reveal any significant differences in the SUMOylation patterns obtained with either ES1 or ES3 compared to the SUMOylation of Ubc9^mut^_TTR (Fig 5B). Moreover, no impact on the SUMOylation patterns from the mutation of lysine residues of TTR was observed, except for K126 (data not shown). The UFDS analysis of the mutants showed that unexpectedly, the SUMOylation of fusion proteins containing TTR and Ubc9 occurred at the Ubc9 portion, likely at more than one lysine residue. The SUMOylation of TTR was negligible in UFDS and might depend on the SUMOylation (SUMO-load) of Ubc9.

To determine whether mutations of Ubc9 lysine residues are reflected in the SUMOylation patterns of other proteins subjected to UFDS, we decided to use the *Drosophila melanogaster* ultraspiracle (Usp). The SUMOylation of Ubc9_Usp has been investigated previously using UFDS and was shown to be attributed to Usp [43]. The SUMOylation patterns of the K14,153,154R mutant of Ubc9 fused to Usp (Ubc9^mut^_Usp) obtained with ES1 or ES3 did not reveal changes in the intensity or mobility of any particular band relative to Ubc9_Usp (S3 Fig). However, we did observe a decrease in the intensity of all of the bands, corresponding to both unmodified and SUMOylated molecules of Ubc9^mut^_Usp, suggesting lower expression or stronger degradation of Ubc9^mut^_Usp compared to Ubc9_Usp.

In conclusion, the SUMOylation of fusion proteins comprising TTR and Ubc9 occurs mainly at the Ubc9 portion, with possible minor modifications of the TTR moiety. The mutation of the lysine residues of Ubc9, which alters the SUMO-load of Ubc9, influences the SUMOylation patterns of fusion proteins. This influence depends on the relative orientation of TTR and Ubc9 and, to a lesser extent than for wild-type Ubc9, on the SUMO isoform.

### Trans-SUMOylation of TTR

**Fig 6:**
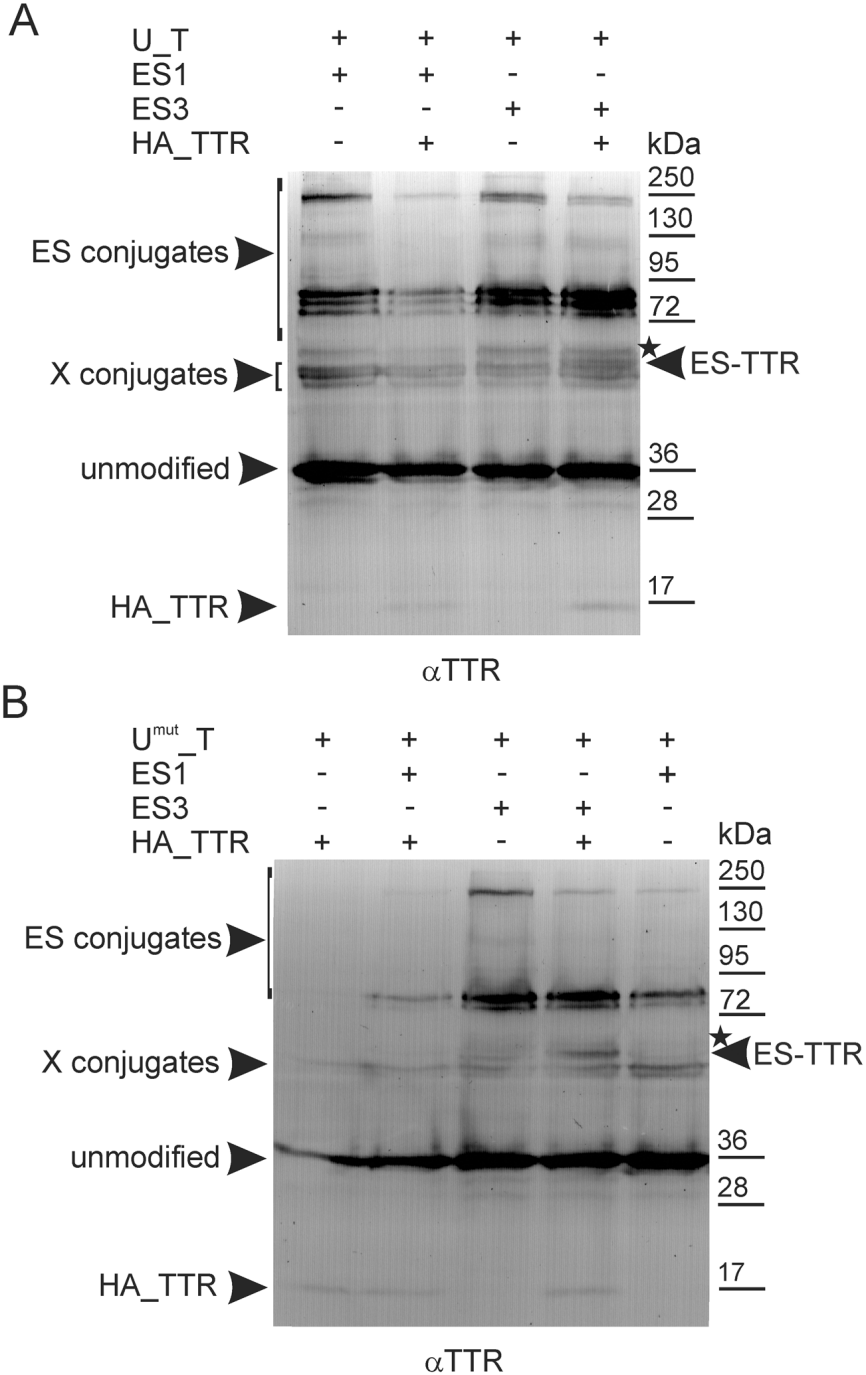
Trans-SUMOylation of non-fused TTR occurs *via* association with Ubc9_TTR or Ubc9^mut^_TTR. TTR fused to the C-terminus of non-mutated (U_T) (A) or the K14,153,154R mutant of Ubc9 (U^mut^_T) (B) was co-expressed with EGFP-labelled SUMO-1 (ES1) or SUMO-3 (ES3) in the absence or presence of non-fused HA-labelled TTR (HA_TTR). The cell lysates were analysed by WB using anti-TTR antibodies (αTTR). The asterisks indicate truncated SUMOylated proteins. ES conjugates and X conjugates represent proteins modified by EGFP-labelled SUMO or endogenous modifiers, respectively.

When we attempted to verify the SUMOylation of non-fused TTR co-expressed with non-fused Ubc9 and ES1 or ES3 in HEK293 cells, TTR was not detected or was detected in WB with the anti-TTR antibodies at a much lower level than that found for any of the fusion proteins (S4 Fig). Consequently, we were unable to determine unequivocally the presence of the products of TTR SUMOylation. Interestingly, the co-expression of Ubc9_TTR (S4 Fig, lanes 1–3), as opposed to non-fused Ubc9 alone or together with EGFP-labelled SUMO (S4 Fig, lanes 4–6), enhanced the TTR signal in WB, suggesting that Ubc9_TTR may interact with and stabilize non-fused TTR. The quaternary structure of TTR is dynamic, which leads to the formation of hybrid tetramers when different TTR monomers are available [2,3,46]. Therefore, we assumed that non-fused TTR might be exchanged with one or more subunits of the Ubc9_TTR tetramer and become SUMOylated when expressed in the same cell. The SUMO-conjugation to the protein occurring because of the association with its binding partner, which is fused to Ubc9, is called trans-SUMOylation and has been successfully used previously to investigate protein-protein interactions [47]. The results of the SUMOylation of non-fused TTR using Ubc9_TTR or Ubc9^mut^_TTR are shown in Fig 6 and S5 Fig. Ubc9_TTR (U_T in Fig 6A and S5A Fig) or Ubc9^mut^_TTR (U^mut^_T in Fig 6B and S5B Fig) co-expressed with ES1 or ES3 produced characteristic patterns composed of their unmodified forms, degradation products (asterisks), endogenous conjugates (X conjugates) and ES1- or ES3-conjugates (ES conjugates) detected with the anti-TTR antibodies in WB. When non-fused TTR was co-expressed with Ubc9_TTR (Fig 6A and S5A Fig lanes +HA_TTR) or Ubc9^mut^_TTR (Fig 6B and S5B Fig, lanes +HA_TTR), one strong band and one weak band were observed, identified collectively as ES-TTR (Fig 6 and S5 Fig). The mobility of ES-TTR corresponded to a MW of 55 kDa, in agreement with the calculated MW of TTR (14.7 kDa) and one molecule of exogenous, EGFP-labelled SUMO (40 kDa). ES-TTR therefore represented SUMOylated, non-fused TTR. Apparently, the trans-SUMOylation of TTR with Ubc9^mut^ _TTR was more efficient than with Ubc9_TTR because the ES-TTR bands were more clearly visible for Ubc9^mut^_TTR than for Ubc9_TTR (Fig 6 and S5 Fig, compare A and B). It must be noted, however, that the detection of non-fused TTR SUMOylated using Ubc9_TTR is obscured by the more abundant products of Ubc9_TTR degradation, which have electrophoretic mobilities similar to that of ES-TTR. When non-fused TTR was co-expressed with fusion proteins possessing TTR located at the N-terminus (TTR_Ubc9 or TTR_Ubc9^mut^), we did not observe bands that were attributable to molecules of SUMOylated, non-fused TTR (data not shown). This inability to support trans-SUMOylation suggests that the location of Ubc9 at the C-terminus of TTR may interfere with the process of subunit exchange [3], which is a prerequisite for trans-SUMOylation. The results of trans-SUMOylation unequivocally confirmed that free TTR (i.e., not fused to Ubc9) could be SUMOylated. Moreover, this process may depend on the SUMO-load of Ubc9, which raises the question of whether TTR, in turn, influences cross- or trans-SUMOylation of Ubc9.

### Dissecting the cross-SUMOylation and trans-SUMOylation of Ubc9 and SUMOylation of TTR

**Fig 7:**
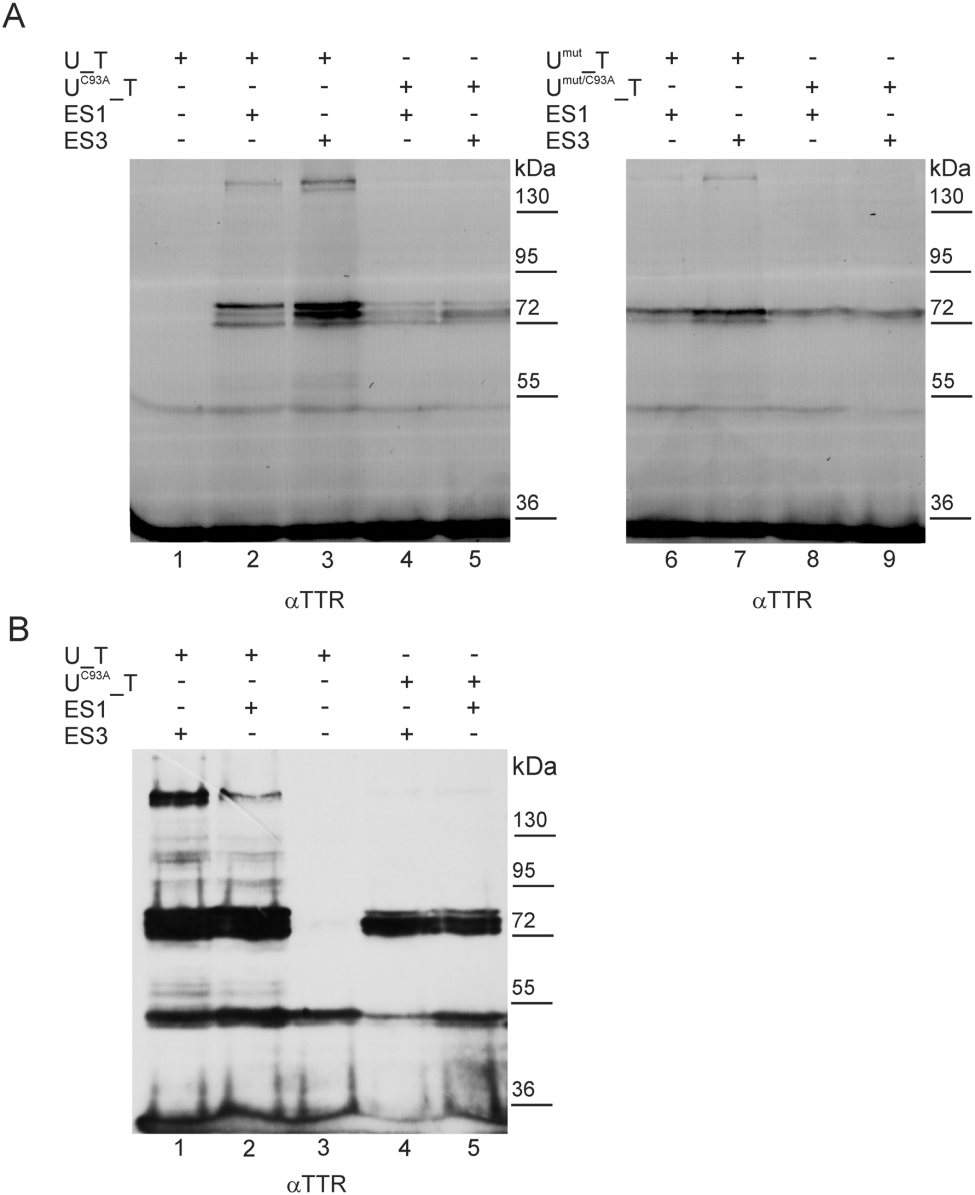
Dissecting cross-SUMOylation and trans-SUMOylation of Ubc9. TTR (T) was fused to the C-terminus of the following Ubc9 forms: non-mutated (U_T), K14,153,154R mutant (U^mut^_T), catalytic site C93A mutant (U^C93A^_T) or mutant harbouring both types of mutations (U^mut/C93A^_T). It was then co-expressed with EGFP-labelled SUMO-1 (ES1) or SUMO-3 (ES3) in HEK293 cells. The cell lysates were analysed by WB using anti-TTR antibodies (αTTR). (A) Fluorescence scans and (B) exposure on photographic film.

To separate the cross-SUMOylation from the trans-SUMOylation of Ubc9 and the SUMOylation of TTR, which were observed concomitantly in the SUMOylation patterns, we mutated the catalytic cysteine 93 to alanine in the wild type and K14,153,154R mutant of Ubc9 and verified the effect of the C93A mutation on the SUMOylation of their respective fusions with TTR (U^C93A^_T and U^mut C93A^_T) using UFDS analysis (Fig 7). Previously, the C93A mutation was shown to eliminate the ability of Ubc9 to transfer SUMO to substrate proteins (trans-SUMOylation) but did not affect direct SUMO-conjugation to Ubc9 lysine residues by the activating enzyme E1 (cross-SUMOylation) [45]. Therefore, we expected that the bands representing the SUMOylation of TTR and the trans-SUMOylation of Ubc9 in the fusion proteins would disappear, whereas the bands representing the cross-SUMOylation of Ubc9 by E1 should be present in the SUMOylation patterns of the C93A mutants of Ubc9 fused to TTR. Our results revealed that mutating the catalytic cysteine dramatically reduced the SUMO-chain-formation abilities of both Ubc9 and Ubc9^mut^ (mutant of lowered SUMO-load capacity) because the HMW bands (above 95 kDa) were completely eliminated (Fig 7B, compare lanes 1, 2 to lanes 4, 5). This effect was stronger than that observed after mutating the lysine residues of Ubc9 (Ubc9^mut^) in fusion proteins (compare Figs 4B and 7B). Additionally, two bands in the MW range of 72 to 95 kDa were significantly weaker than those observed for Ubc9_TTR. However, all three bands remained present in the SUMOylation patterns of Ubc9^C93A^_TTR (Fig 7A compare lane 2 to 4 and lane 3 to 5). The mutation of C93A in Ubc9^mut^ fused to TTR also reduced SUMOylation (Fig 7, lanes 6–9). The HMW conjugates were absent, and only one band, in the MW range of 72 to 95 kDa, which represents mono-SUMOylated Ubc9^mut/C93A^_TTR, appeared to be present. These data show that the presence of catalytic C93 of Ubc9 is strictly required for poly- or multi-SUMOylation. Because mono-SUMOylation occurs even when Ubc9 fused with TTR does not possess catalytic C93, we conclude that the lysine residues of Ubc9 are cross-SUMOylated by E1. Interestingly, the patterns of cross-SUMOylation resemble those obtained for non-mutated fusion proteins (in the MW range of 72–95 kDa), *s*uggesting that the same lysine residues accept SUMO molecules from E1 (cross-SUMOylation) and Ubc9 (trans-SUMOylation). Although this observation also suggests that SUMOylation occurs mainly at the lysine residues of Ubc9 fused to TTR, the possibility that a small percentage of SUMO molecules are conjugated to the TTR portion cannot be ignored. The mobility of the TTR SUMOylation products must then overlap with the mobility of the fusion proteins SUMOylated at Ubc9 lysine residues. For fusion proteins composed of TTR and the C93A mutant of Ubc9^mut^ with lower SUMO-load capacity (U^mut C93A^_T in Fig 7), only one of two bands in the MW range of 72 to 95 kDa appears to be present (Fig 7, compare lane 6 to 8 and lane 7 to 9), indicating that one lysine residue of Ubc9^mut^ is cross-SUMOylated and that another lysine residue of Ubc9^mut^ or lysine residue of TTR is trans-SUMOylated.

To identify the positions of the SUMOylated lysine residues in the fusion proteins analysed by UFDS, we employed MS analysis. A T95R mutant of SUMO 1 labelled with EGFP allowed us to identify a di-glycine motif in MS at the SUMOylation sites after tryptic digestion of SUMOylated proteins. We analysed the SUMOylation sites in Ubc9_TTR because this fusion protein displayed the strongest SUMOylation signal in UFDS. The samples were prepared for MS analysis according to two alternative protocols. The first method consisted of using SDS-PAGE to resolve the whole cell extracts obtained by UFDS and then cutting out the piece of gel containing the SUMOylation products in the MW range of 72–95 kDa. The second approach comprised immunoprecipitating the SUMOylated fusion proteins with anti-TTR antibodies from the cell extracts obtained by UFDS. MS analysis identified K154 of Ubc9 as the primary SUMOylation site in Ubc9_TTR, regardless of the sample-preparation mode. When the MS samples were prepared by SDS-PAGE, we also found peptides corresponding to Ubc9 SUMOylated at K18. The immunoprecipitation method allowed us to identify K49 and K65 as SUMOylation sites of Ubc9. Interestingly, we did not find peptides SUMOylated at K14 or K153 of Ubc9, although these sites have been identified as the primary SUMOylation sites in non-fused Ubc9, with K14 being essential for the SUMOylation specificity of Ubc9 [44,45]. Using MS, we were also unable to identify any peptide with a TTR lysine residue modified with SUMO.

### The influence of TTR on global SUMOylation

**Fig 8:**
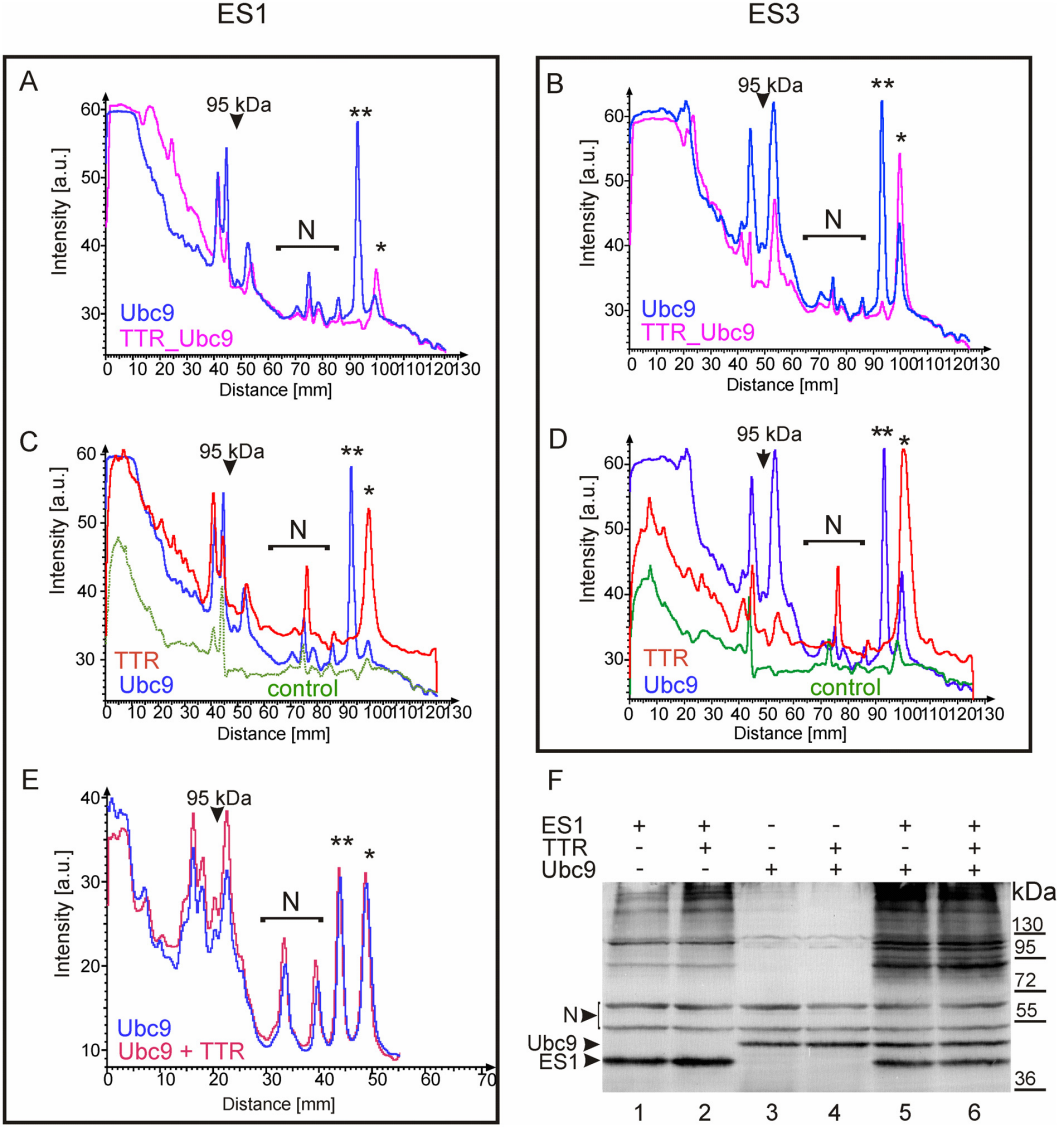
Non-fused and Ubc9-fused TTR influences general SUMOylation. Each of protein—non-fused TTR (TTR), TTR fused to the N-terminus of Ubc9 (TTR_Ubc9), and non-fused Ubc9 or EGFP (control)—was co-expressed with EGFP-labelled SUMO-1 (ES1) or SUMO-3 (ES3) in HEK293 cells. The cell lysates were analysed by WB using anti**-**GFP antibodies. The histograms present the intensities of the bands measured in the lanes containing the lysates of the cells expressing the indicated proteins. The intensity of each band is plotted against its distance from the top of the separating gel (marked with 0). The superimposed histograms represent the samples run on the same gel (A, B and E) or on parallel gels (C and D). The arrows represent the position of the 95-kDa protein marker. The asterisks indicate free ES1 and ES3; the double asterisks show the Ubc9 bands. N marks the region containing unspecific bands.

The SUMOylation of the fusion protein composed of TTR and C93A mutant of Ubc9 resembled the SUMOylation of the non-fused C93A Ubc9 mutant in the presence of Rhes, which is a SUMO ligase for Ubc9 [45]. Moreover, SUMOylation has been found to influence substrate specificity of Ubc9 [33,44,45] and to play a regulatory role in the SUMO pathway. Therefore, we aimed to verify whether the impact of TTR on the SUMOylation of Ubc9 has consequences for global SUMOylation. Thus, we expressed ES1 or ES3 in HEK293 cells and observed the effect of the co-expression of TTR, Ubc9 or TTR_Ubc9 on SUMO-conjugation to cellular proteins by WB using antibodies directed against the exogenous SUMO tag (EGFP). The signal intensity was densitometrically determined in each lane with AIDA Image Analyser software, and plotted against the position relative to the top of the separating gel (Fig 8A–8E). The quantity of HMW SUMO-conjugates of cellular proteins was higher in the presence of TTR co-expressed with ES1 (Fig 8C and 8F, compare lane 1 to 2) or, to a lesser extent, ES3 (Fig 8D). We also observed an increase in global SUMOylation induced by Ubc9 (Fig 8C) which agreed with the previous results obtained using Ubc9 transgenic mice [48]. However, the general SUMOylation patterns appear to be different for TTR and Ubc9 in terms of not only the profile shapes but also the effect of the particular SUMO isoform (Fig 8, compare C to D). In our experimental system, if the expression levels of ES1 or ES3 were moderate, Ubc9 exerted stronger effects than TTR regarding ES3-conjugates with HMW or MW close to 95 kDa (Fig 8D). In contrast, the increase in ES1-conjugates was slightly stronger, or at least similar, in the presence of TTR compared to Ubc9, and the overall profiles were quite similar (Fig 8C). Discriminating between the effects of TTR and Ubc9 could also be achieved by increasing the ES1 expression level (over twofold) because general SUMOylation was further elevated in the presence of Ubc9 compared to that resulting from TTR, which appeared to reach saturation (Fig 8F, compare lane 1 to 2 and 5). Interestingly, the SUMO-conjugation to cellular proteins in the presence of TTR and Ubc9 together was not equal to the sum of the effects induced by TTR and Ubc9 individually. The co-expression of TTR and Ubc9 did not simply increase the ES1-conjugation evoked by Ubc9 (or TTR), and the amount of the HMW conjugates was even slightly lower than for Ubc9 alone (Fig 8E and 8F, compare lane 5 to 6). The impact of the fusion protein TTR_Ubc9 on global ES1-conjugation and ES3-conjugation (Fig 8A and 8B) was a unique combination of the effects observed for non-fused TTR or Ubc9. An even greater increase in ES1-conjugation was caused by TTR_Ubc9 than by TTR alone, exceeding that exerted by Ubc9 (Fig 8A and 8C). In the case of ES3-conjugation, the SUMOylation profile of TTR_Ubc9 followed that induced by Ubc9 except for MWs of approximately 95 kDa (Fig 8B). Interestingly, TTR seemed to protect ES1 (and, to a lesser extent, ES3) against degradation, as shown by the levels of free ES1 in Fig 8C (asterisks). This observation may seem counterintuitive, but a simultaneous increase in the amounts of conjugated and free SUMO has been previously reported [49]. Our data indicate that TTR regulates general SUMOylation in a complex manner.

## Discussion

Ongoing intensive research aims to understand the role of PTMs in the molecular pathogenesis of protein-misfolding diseases [28,30,35,36,50,51]. Accumulating data indicate that proteins associated with amyloid diseases are subjected to extensive, “cross-talking” PTMs [33,35,39]. Specifically, α-synuclein, tau, huntingtin, amyloid precursor protein, DJ-1 and other amyloidogenic proteins have been shown to be SUMOylated [30]. Therefore, we decided to verify whether TTR is also a SUMOylation substrate. We chose UFDS because it facilitates enhanced detection of SUMOylation products. Moreover, UFDS, by fusing Ubc9 to the investigated protein, imitates the natural contact between the substrate and SUMOylation enzymes. Thus, UFDS seems to be more suitable for investigating SUMOylation than *in vivo* or *in vitro* systems in which the elements of the SUMOylation reaction are separately expressed or added. The close contact between the enzymes of the SUMOylation machinery and the substrate *in vivo* is a prerequisite for SUMO-conjugation and, hence, the existence of SUMO ligases and SUMO-interacting motifs [31,33,34]. It should be noted that the covalent linkage of Ubc9 to the analysed protein should not induce SUMOylation. Recently, X-ray crystallography revealed the presence of a Ubc9 homodimer, indicating that Ubc9 molecules interact with each other [52]. Therefore, the specificity of the SUMO-conjugation to the substrate protein should not be dependent on the homotypic interactions of Ubc9 molecules. Although we cannot entirely exclude that bringing together Ubc9 moieties through tetramerization of the TTR portion of fusion proteins facilitates contact between two Ubc9 molecules, the following findings suggest that our observations have physiological meaning and are not simply induced by Ubc9-Ubc9 interactions: 1. the occurrence of the trans-SUMOylation of non-fused TTR; 2. similar SUMOylation patterns for Ubc9 and the catalytic site mutant of Ubc9 fused to TTR, differing mainly in the presence of poly-SUMOylated forms; 3. the unique set of SUMOylated lysine residues of Ubc9 in Ubc9_TTR compared to SUMOylation sites observed for non-fused Ubc9 [38,44,45]; 4. distinct effects exerted on general SUMOylation by TTR, Ubc9 and fusion proteins; and 5. the apparent dependence of SUMOylation of TTR on the SUMO-load of Ubc9.

*In vivo*, TTR functions as a tetramer, and only a small fraction of its molecules have been found to dissociate into monomers [2,46], which is considered an initial step in fibrillogenesis [53]. Our data show that fusion proteins composed of TTR and Ubc9 form tetramers. The Ubc9 and TTR portions of the fusion proteins are separated by five-amino acid spacers that do not contain lysine residues. Given that TTR is a dimer of dimers and that both the N- and C-termini of each monomer are directed outward, two Ubc9 moieties should locate at each side of the TTR tetramer with no apparent steric hindrance. In Ubc9_TTR, the disordered N-terminus of TTR provides additional freedom to the Ubc9 moiety [2,54,55].

Analysing TTR_Ubc9 and Ubc9_TTR in the UFDS system revealed the formation of multiple exogenous SUMO-conjugates of different MWs ranging from 72 to more than 250 kDa. Up to twenty bands, which could be attributed to the SUMOylation products, were detected after probing WB with anti-TTR antibodies, followed by prolonged exposure on photographic film (S6 Fig). Thus, SUMOylation may have occurred at multiple lysine residues, producing differently branched polypeptides with distinct electrophoretic mobilities. The sub-stoichiometric amounts of some protein forms in the SUMOylation patterns suggest the presence of multiple SUMO acceptor sites that differ in their availability for SUMO-conjugation. Alternatively, sub-populations of SUMO-modified molecules may be generated as a result of other PTMs. For example, mixed SUMO-ubiquitin chains or mixed SUMO chains composed of EGFP-labelled SUMO and endogenous modifiers might be formed. Many other proteins are regulated through the complex interplay of various PTMs [33–35]. Moreover, SUMOylation and other cross-talking PTMs may occur at residues other than lysine. In the SUMOylation patterns, some faint HMW bands were less visible in replicate experiments, suggesting that PTMs might depend on variations in cell growth [35,56] or the activity of deSUMOylating enzymes.

Our data showed that the arrangement of TTR and Ubc9 in the fusion proteins influenced the SUMOylation patterns. The observed dissimilarities could result from the fact that Ubc9_TTR may be more accessible to E1 than TTR_Ubc9 because E1 interacts with the N-terminal ubiquitin fold domain of Ubc9 [57]. Moreover, the accessibility of the N- or C-terminal sequences to relevant PTMs often depends on protein-protein interactions in the cell; therefore, a strong relationship exists between PTMs and protein localization [30,31,35,51]. In particular, SUMOylation occurs inside the cell *in situ* (e.g., in promyelocytic leukaemia bodies) within the functional and structural compartments of the nucleus, the cytoplasm or the membranes [31,34,58,59]. TTR and Ubc9 have distinct predominant localizations: partially similar and partially dissimilar. Ubc9 can be found in the cytoplasm and the nucleus [60], predominantly in association with nuclear bodies [58,61,62], the nuclear envelope [60] or the filaments of the nuclear pore complex [59]. Ubc9 has also been found within the lumen of the endoplasmic reticulum [63]. TTR has been observed in the membrane and the nuclear and mitochondrial compartments [10]. In ependymal cells, TTR is localized in the cytoplasm, the cilia and around the mitochondria, probably associated with the outer mitochondrial membrane [7]. To perform trans-SUMOylation experiments, we utilized the ability of TTR to combine different subunits into a complex [3]. We observed SUMO-conjugation to non-fused TTR only when Ubc9_TTR, and not TTR_Ubc9, was used. Similarly, different localizations of TTR_Ubc9 and Ubc9_TTR or different cellular partners might result in localization-specific interactions that inhibit the process of subunits being swapped in the complex, as observed for TTR in the presence of retinol-binding protein [3].

Our data, which were obtained *via* site-directed mutagenesis and UFDS analysis, showed that the mobility of one of three bands in the 72–95 kDa MW range changed only for the K126R mutant of TTR fused to the N-terminus of Ubc9 (TTR^126^_Ubc9). However, we did not observe an effect of the K126R mutation of TTR fused to the C-terminus of Ubc9 (Ubc9_TTR^126^). K126 may be inaccessible to SUMOylation in Ubc9_TTR. Furthermore, the K126R mutation in TTR_Ubc9 may alter the position of the sites of SUMOylation or other PTMs in the TTR or Ubc9 portion of the fusion protein. When the wild-type Ubc9 was exchanged with the K14,153,154R mutant (U^mut^) with decreased SUMO-load capacity, we observed that a few weak bands disappeared from the SUMOylation patterns, suggesting that SUMO-conjugation to TTR lysine residues can occur in a very small population of molecules. This observation would explain why we were unable to detected SUMOylated TTR peptides using MS. Mutating K154, K153 and K14 in Ubc9 fused to TTR strongly affected the SUMOylation patterns, indicating that the SUMOylation of Ubc9 overcomes the SUMOylation of TTR in the UFDS system. The MS data showed that Ubc9 fused to TTR is predominantly SUMOylated at K154 (ion score equal to 84, S7 Fig), in agreement with our site-directed mutagenesis results. Surprisingly, we did not find SUMOylated peptides at the adjacent, previously identified K153 [45] or at K14, which has been shown to be the major SUMOylation site in human Ubc9 [44,45]. However, in Ubc9 fused to TTR, we observed the SUMOylation of K49, which has been reported for non-fused Ubc9 [45]. We also found unique and previously undetected SUMOylation of K18 and K65 of Ubc9 in Ubc9_TTR. SUMOylation of K18 in Ubc9_TTR is particularly interesting because this residue is involved in the interaction of Ubc9 with SUMO [64]. Moreover, the ubiquitination of K18 has been observed in murine Ubc9 [65]. Intriguingly, the SUMOylation of K65 was also noted; interestingly, the acetylation of K65 was recently shown to determine the substrate specificity of human Ubc9 [66]. Previously, the substrate preference of Ubc9 toward a sub-set of cellular proteins was found to depend on the SUMOylation of K14 [44], and mutating K14, K49 and K153 of Ubc9 to arginine residues significantly reduced the SUMOylation of SP100, resulting in slight changes in SUMOylation of RanGAP and IκB [45]. We observed that the SUMOylation of a unique set of lysine residues of Ubc9 depended on fusion with TTR. Two of these residues, K18 and K65, are located at a crossroads between SUMOylation and other PTMs: K18 may undergo ubiquitination, and K65 was acetylated in non-fused Ubc9. If TTR determines the preference for SUMOylation over acetylation at K65 or SUMOylation over ubiquitination at K18 of Ubc9, it must impose a very strong regulatory effect.

Additionally, the SUMOylation of TTR and Ubc9 appears to be interconnected. We found that mutating the catalytic cysteine 93 of Ubc9 to alanine resulted in the disappearance of nearly all HMW species, suggesting that SUMO-chains are formed at Ubc9 or TTR lysine residues *via* trans-SUMOylation. In yeast, SUMOylated (SUMO-loaded) Ubc9 has been shown to exhibit impaired classical SUMO-conjugating activity and instead acts as a scaffold to promote SUMO-chain formation [67,68]. If a similar relationship occurs in humans, Ubc9 fused to TTR should be SUMO-loaded, which is in agreement with our MS and site-directed mutagenesis results. Consequently, the SUMO-conjugating activity of Ubc9 toward the substrate (TTR) would be diminished. The SUMOylation of TTR fused to Ubc9, therefore, depends on the SUMO-load of Ubc9. Moreover, the SUMOylation of lysine residues of Ubc9 influences its substrate specificity [33,44,45], and the SUMOylation of TTR may be altered and possibly reduced, which would be in agreement with the clearly visible trans-SUMOylation of TTR by a Ubc9 mutant with impaired SUMO-load capacity. It is possible that for efficient SUMOylation of TTR, Ubc9 should not be in the SUMOylated form.

At least part of the observed impact of TTR on global SUMOylation may be attributed to the influence of TTR on the SUMOylation of Ubc9 and, thus, on the specificity of SUMO-conjugation by Ubc9 to cellular proteins. The SUMO-load of Ubc9 strongly affects the SUMOylation of proteins containing the SUMO Interactive Motif [33,69]. Therefore, TTR may exert a unique and profound effect on downstream signalling within the cell, influencing SUMOylation and, consequently, the fate of numerous target proteins. One intriguing question is whether the regulatory activity of TTR regarding SUMO-conjugation to Ubc9 requires TTR to be SUMOylated or not. In other words, does the SUMOylation of TTR affect the SUMOylation of Ubc9 and global protein SUMOylation?

A complex relationship exists between the SUMOylation of Ubc9 and E1. E1 is auto-SUMOylated on K236 and SUMOylated by Ubc9 on multiple lysine residues [45], which results in the repression of the SUMO transfer from E1 to the catalytic cysteine of Ubc9 and then to substrate proteins [70]. This relationship constitutes a negative regulatory loop in the SUMOylation process [70]. After heat shock, the SUMOylation of E1 is reduced, abolishing the suppression of SUMO-conjugation [70]. By influencing the SUMOylation of Ubc9 and leading to a unique set of SUMOylated lysine residues in Ubc9, TTR may serve as a modulator of the SUMOylation reaction. Similar to TTR, the exogenous expression of Toll interacting protein (Tollip) activates the SUMOylation of cellular proteins in 293T cells [71]. Tollip is SUMOylated and interacts with proteins involved in the SUMOylation process, including Ubc9, suggesting that Tollip functions as a SUMO ligase. Tollip’s involvement in the PTM system however, is complex and appears to extend far beyond this activity [71]. Rhes also increases the cross-SUMOylation of Ubc9 and has been reported to fulfil the criteria for the E3 of Ubc9 [45]. The knockout of Rhes diminished the global SUMOylation in the striatum [45], suggesting that it positively influences global SUMOylation and confirming the similarity between TTR and Rhes. In the context of a compromised heat-shock response, TTR was shown to contribute to controlling neuronal cell death, oedema and inflammation in the brain [72]. Surprisingly, the brains of TTR^(-/-)^ null mice showed no apparent susceptibility to ischaemic damage. However, in mice heterozygous for HSF1 ^(+/-)^, the knockout of TTR and middle cerebral artery occlusion (MCAO) induced particularly severe injuries [72]. The unexpectedly profound effect of TTR depletion in mice partially deprived of the response to oxidative stress may be at least partially explained by the influence of TTR on the SUMOylation of Ubc9, which should alter the substrate specificity of Ubc9 toward cellular proteins. The observation that increased SUMOylation, mainly SUMO-1 but also SUMO-2/3-conjugation, protected the brain against ischaemia induced by MCAO in Ubc9 knock-in-mice supports this hypothesis [34,48]. However, although the cerebral ischaemia induced in mice brains was accompanied by a massive increase in protein SUMO-2/3-conjugation, a transient decrease in the level of Ubc9 was observed in the cortex [73]. This apparent paradox may result from the presence of a limiting factor other than Ubc9 able to modulate global protein SUMOylation [73]. TTR is a good candidate for being this factor because TTR affects global protein SUMOylation, unlike Ubc9, exhibits a preference for SUMO-1-conjugation and is distributed throughout the infarct [72]. TTR may be involved in controlling more than just the stress-response because SUMOylation regulates diverse cellular processes [31,34]. TTR appears to influence both SUMO-3, a major stress-induced SUMO isoform, and SUMO-1-conjugation. The SUMO cascade, which is conserved among all eukaryotes, regulates protein localization, transcription, DNA replication, and mitosis [34]. TTR may be indirectly engaged in all of these processes by influencing Ubc9 substrate specificity and global protein SUMOylation. An exciting but still unverified discovery was the interaction of TTR with IKBKAP, a subunit of the α-tubulin acetyltransferase (Elongator) complex [74]. Correspondingly, the absence of TTR delayed crushed nerve regeneration due to slower retrograde transport [17]; this effect may be related to the impaired acetylation of α-tubulin by the Elongator complex and consequent dis-regulation of intracellular transport, which appears to be the underlying mechanism of various neurological disorders [75].

Although the involved proteins and physiological symptoms are different in particular diseases, the primary source of a range of pathological states, including cancer, appears to be an imbalance in the folding and degradation pathways, which may be caused by the dis-regulation of the PTMs of cellular proteins throughout the whole proteome [29,76]. Thus, factors that control PTMs could influence the wellness of the entire body. The more up-stream a particular factor functions in the regulatory circuits, the more profound an effect it should exert. TTR, by influencing the specificity of Ubc9 and regulating the SUMOylation of cellular proteins, is a good candidate as a wellness-protection factor.

## Conclusions

The SUMOylation of TTR in the UFDS system shows multiple SUMOylation sites (Fig 2). Unusually, SUMOylation occurs mainly at the Ubc9 portion, with possible minor modifications of TTR (Figs 3–5). Importantly, Ubc9 fused to TTR is SUMOylated at a unique set of lysine residues, that are distinct from the known SUMOylation sites of the non-fused Ubc9. Non-fused TTR is SUMOylated *via* trans-SUMOylation by Ubc9 fused to TTR (Fig 6). Preliminary experiments indicate that TTR specifically modulates general protein SUMOylation (Fig 8).

## Materials and Methods

### Plasmid construction

The cDNAs of wild-type and mutants of TTR devoid of the sequence-coding signal peptide were amplified by polymerase chain reaction (PCR) and cloned into pcDNA-Ubc9-MCS, pcDNA-Ubc9^mut^-MCS, pcDNA-MCS-Ubc9 or pcDNA-MCS-Ubc9^mut^ expression vectors suitable for eukaryotic cell lines. Ubc9^mut^ represents the K14,153,154R mutant of Ubc9. Mutants of TTR were generated using a PCR-based mutagenesis protocol [77]. The constructs coding TTR non-fused to Ubc9 were obtained by exchanging the cDNA of Ubc9 in the pcDNA-Ubc9-MCS vector with the cDNA of TTR with a haemagglutinin tag obtained by PCR. The constructs of ES1, ES3 and EYFP-labelled Ubc9 were described previously [41]. The cDNA of rTTR possessing a C-terminal histidine tag was cloned into the pGEX-2T vector for protein expression in *Escherichia coli*.

### Cell culture and transfection

HEK293 cells were cultured in Dulbecco's modified Eagle´s medium containing high glucose and stable glutamine (PAA, E15-883), 10% foetal calf serum (PAA, A15-152), 100-U/ml penicillin and 100-μg/ml streptomycin (PAA, P11-010). Subsequently, 60–80%-confluent HEK293 cells were transfected with polyethylenimine and grown for 24 h. Unless stated otherwise, the cells were lysed in a Laemmli gel loading buffer [78] with or without heat denaturation for 10 min at 95°C and then stored at -80°C.

### Western blotting

Samples of cell lysate proteins were denatured (typically for 5–10 min at 95°C or at the indicated conditions) immediately before loading on an SDS gel and undergoing separation in a Laemmli buffer system and transfer to a nitrocellulose membrane (Whatman, 10401196). The primary antibodies anti-Ubc9 (Santa Cruz, sc-271057), anti-GFP (Clontech, 632376) and anti-TTR (ProteinTech, 11891-1-AP), the horseradish peroxidase-conjugated secondary antibody (Jackson ImmunoResearch, 111-035-045) and the chemiluminescence reagents (GE Healthcare, Amersham RPN2232) were used according to the manufacturers’ instructions. The chemiluminescence intensity was measured using Fluorescent Image Analyser FLA-3000 (Fujifilm) and AIDA Image Analyser software (Raytest Isotopenmeßgeräte GmbH, Straubenhardt, Germany) or detected using photographic film (Kodak). The MW was determined using Prestained Protein Leader Plus (Fermentas, SM1811), PageRuler^™^ Prestained Protein Ladder (Thermo Fisher Scientific, 26616) or Unstained Protein Molecular Weight Marker (Fermentas, SM043).

### *In silico* analysis

The amino acid sequences of human TTR (UniProtKB, P02766) and Ubc9 (UniProtKB, P63279) were analysed using the following bioinformatic tools: Sumo sp (http://sumosp.biocuckoo.org/), Sumo plot (http://www.abgent.com/tools/), and PCI-Sumo (http://bioinf.sce.carleton.ca/SUMO/start.php).

### TTR expression and purification in *Escherichia coli*

Recombinant TTR (rTTR) fused with glutathione-S-transferase was expressed in the *E*. *coli* BL21 pLys cell line. After inducing protein expression with 1-mM isopropyl β-D-1-thiogalactopyranoside (IPTG), the cells were cultured for 3 h at 37°C, harvested by centrifugation, washed and suspended in phosphate-buffered saline (PBS) containing 1-mM dithiotreitol (DTT) and frozen at -80°C. Prior to purification, the cell suspension was thawed, and the cells were lysed by pipetting. DNase I, RNAse A and phenylmethylsulfonyl fluoride were added to final concentrations of 10 μg/ml, 10 μg/ml and 0.2 μg/ml, respectively. The soluble fraction was obtained by centrifugation for at least 1 h at 18000xg and was loaded on a glutathione-sepharose column equilibrated with PBS. Contaminating proteins were washed out with PBS, and rTTR was digested out of the glutathione-S-transferase with thrombin (Calbiochem) at room temperature for up to 24 h. rTTR-containing fractions were collected by washing the column with PBS, concentrated using a centricon (Millipore), loaded onto a Superdex 200 column (GE Healthcare, 17-5175-01) and separated using Akta Explorer (Amersham Biosciences).

### Sedimentation velocity analysis

Sedimentation velocity experiments were conducted in a Beckman Coulter ProteomeLab XL-I ultracentrifuge (Software version 6.0, Beckman Coulter Inc.). An-60Ti rotor and cells with sector-shaped 2-channel charcoal filled Epon^®^ centrepieces were used. The sample sector was filled with 400 μl of 0.5-mg/ml TTR in 9-mM 2-[4-(2-hydroxyethyl)piperazin-1-yl]ethanesulfonic acid (HEPES), and 92-mM NaCl at pH 7.3. The reference sector contained 405 μl of the same buffer. The experiment was conducted overnight at 42,000 rpm at 20°C. TTR sedimentation was observed by recording the absorbance at 280 nm.

The data analysis was performed using SEDFIT (available at http://www.analyticalultracentrifugation.com/). Scans representing the whole sedimentation process were selected for data analysis. The density (ρ = 1.0027 g/ml) and dynamic viscosity (η = 1.0192 mPa·s) of the buffer and the partial specific volume of TTR at 20°C (ῡ = 0.72784 ml/g) were estimated using SEDNTERP (available at http://sednterp.unh.edu/). A timestamp correction was made using a function implemented in SEDFIT [79]. A continuous c(s) distribution model was used to obtain the values of the sedimentation coefficient (s) and frictional ratio (f/f_0_). The hydrodynamic radius (R_H_) and apparent MW of TTR were also calculated. Maximum entropy regularization with p = 0.95 was applied [80].

### Sample preparation for MS

Protein samples were prepared for MS using two alternative methods. The first method included SDS-PAGE of the HEK293 cell lysate proteins obtained after UFDS. The gels were briefly stained with Coomassie blue R250, and the appropriate regions of the gel were cut out, transferred to the Eppendorf tubes and de-stained with 10% acetic acid and 40% methanol. Afterwards, a standard procedure for sample preparation for LC-MS/MS analysis was performed [81]. Briefly, proteins were reduced, alkylated, and digested with trypsin and peptides were extracted from the gel. The second protocol was performed according to [82] with some modifications. HEK293 cells obtained after UFDS were collected, washed with PBS and lysed in PBS containing 0.1% NP40 and 20-mM N-ethylmaleimide (Sigma) for 30 min at 37°C. After centrifugation for 60 min at 20000xg and 4°C, the soluble proteins were incubated with appropriate amounts of polyclonal anti-TTR antibodies (Dako, A0002) for 1 h at 4°C. The complexes of antibodies and TTR-containing fusion proteins were collected by centrifugation for 15 min at 20 000xg and 4°C, washed at least 3 times with PBS and vacuum dried. Prior to MS analysis, the protein samples were suspended in 0.1-M ammonium bicarbonate buffer containing 6-M urea. Proteins were reduced for 15 min in 20-mM DTT and alkylated for 20 min in the dark in 60-mM iodoacetamide. Next, typically, 1 μg of Trypsin/Lys-C Mix (Mass Spec Grade, Promega, Madison, WI) was added. After 4 h of incubation at 37°C, the samples were diluted with 0.05-M ammonium bicarbonate buffer to a final concentration of 1-M urea, and incubation was continued overnight at 37°C. Digestion was terminated by adding trifluoroacetic acid (TFA) to a final concentration of 1%. The peptide mixture was cleaned using reversed-phase C18 StageTips made in-house [83].

### Liquid chromatography and tandem MS (LC-MS/MS)

MS analysis was performed by LC-MS/MS on a Q-Exactive MS instrument (Thermo Fisher Scientific) equipped with a Digital PicoView 550 nanospray source (New Objective) coupled to an UltiMate 3000RS LC nanoSystem (Thermo-Dionex). Peptides were injected into a C18 precolumn (AcclaimPepMap100, 2 cm x 75 μm inner diameter, C18, 3 μm, 100 Å) using 2% acetonitrile with 0.05% TFA as the mobile phase and further separated on an analytical column (AcclaimPepMapRLSC, 15 cm × 75 μm, C18, 2 μm, 100 Å) with a 2–40% ACN gradient in 0.05% formic acid for 60 min. In the first run, the instrument was operated in data-dependent mode with an inclusion list of selected precursors, and in the second run, it was operated in targeted mode (tMS^2^ mode). The list of precursors derived from the possible Ubc9_TTR peptides with a GlyGly-tagged lysine was generated by the ProteinProspector tool (http://prospector.ucsf.edu/prospector/mshome.htm). The MS/MS spectra were acquired in the Orbitrap mass analyser with a resolution of 35,000 at m/z 200 with an isolation window of 1.2 m/z, a target value of 5.00E+05 and a maximum injection time of 120 ms.

### MS data analysis

The RAW files were processed by the Proteome Discoverer platform (v. 1.4, Thermo Fisher Scientific) and searched against the cRAP database (132 sequences) with a Ubc9_TTR protein sequence The searches were performed using an in-house MASCOT server (v. 2.5.1, Matrix Science) with the following parameters: enzyme, trypsin; maximum number of missed cleavages, 2; fixed modification, carbamidomethylation (C); variable modifications, oxidation (M) and GlyGly-tagged (K); peptide mass tolerance, ±10 ppm; and fragment mass tolerance, ±20 mmu. The mass spectrometry proteomics data have been deposited to the ProteomeXchange Consortium *via* the PRIDE [84] partner repository with the dataset identifier PXD004515.

## Supporting Information

S1 Fig Purity of recombinant TTR. The sample containing 10 μg of recombinant TTR possessing a C-terminal histidine tag (rTTR), purified as described in the Materials and Methods, was heated in Laemmli gel loading buffer for 30 min at 95°C and loaded on 12% SDS gel. After electrophoresis the gel was stained with Coomassie blue R250.
(TIF)

S2 Fig K126R mutation of TTR in TTR_Ubc9 alters the SUMOylation pattern. Non-mutated TTR_Ubc9 (T_U) or the K126R mutant of TTR in TTR^126^_Ubc9 (T^126^_U) were co-expressed in HEK293 cells with EGFP-labelled SUMO-1 (ES1) or SUMO-3 (ES3). The cell lysates were analysed by WB using anti-TTR antibodies (αTTR). The outlined areas have been enlarged. The letters indicate changes in the mobility of the bands resulting from the K126R mutation in TTR. ES conjugates and X conjugates indicate forms of the fusion proteins modified by EGFP-labelled SUMO or endogenous modifiers, respectively.
(TIF)

S3 Fig Mutating the lysine residues of Ubc9 in Ubc9_USP does not affect the SUMOylation patterns. *Drosophila melanogaster* Ultraspiracle protein (USP) fused to non-mutated (Ubc9) or to the K14,153,154R mutant of Ubc9 (Ubc9^mut^) located at the C-terminus (Ubc9_USP and Ubc9^mut^_USP) was co-expressed with EGFP-labelled SUMO-1 (ES1), SUMO-3 (ES3) or EGFP (E) in the HEK293 cell line. Cell lysates were analysed by WB using anti-Ubc9 antibodies (αUbc9). ES conjugates and X conjugates indicate proteins modified by EGFP-labelled SUMO or endogenous modifiers, respectively.
(TIF)

S4 Fig The Ubc9_TTR fusion protein enhances the stability of non-fused TTR. Non-fused HA-labelled TTR (HA_TTR) was co-expressed with EGFP-labelled SUMO-1 (ES1), SUMO-3 (ES3), EGFP (E) or EYFP-labelled Ubc9 (Y_U) in the absence or presence of TTR fused to the C-terminus of Ubc9 (U_T). The cell lysates were analysed by WB using anti-TTR antibodies (αTTR). ES conjugates and X conjugates indicate forms of the fusion proteins modified by EGFP-labelled SUMO or endogenous modifiers, respectively.
(TIF)

S5 Fig Trans-SUMOylation of non-fused TTR. Exposure on photographic film of (A) the WB presented in Fig 6A and (B) WB performed using equivalent samples of the same transfection experiment as presented in Fig 6B. TTR fused to the C-terminus of non-mutated (U_T) (A) or the K14,153,154R mutant of Ubc9 (U^mut^_T) (B) was co-expressed with EGFP-labelled SUMO-1 (ES1) or SUMO-3 (ES3) in the absence or presence of non-fused HA-labelled TTR (HA_TTR). The cell lysates were analysed by WB using anti-TTR antibodies (αTTR). The asterisks indicate truncated SUMOylated proteins. ES conjugates and X conjugates represent proteins modified by EGFP-labelled SUMO or endogenous modifiers, respectively.
(TIF)

S6 Fig The Ubc9_TTR fusion protein is extensively SUMOylated in UFDS. TTR fused to the C-terminus of Ubc9 (U_T) was co-expressed in HEK293 cells with EGFP-labelled SUMO-1 (ES1), SUMO-3 (ES3) or EGFP (E). Cell lysates were analysed by WB using anti-TTR antibodies (αTTR). (A) Fluorescence scans and (B) exposure on photographic film.
(TIF)

S7 Fig Fragmentation spectra of the SUMOylated peptide of Ubc9_TTR. Annotated MS/MS spectra of the precursor ion at *m/z* 962.955 (2+) correspond to peptide **k(GG)**FAPSEFGGGGPTGTGESK. The peptide was assigned to the MS/MS spectrum with a MASCOT ion score of 84. The mass spectrometry proteomics data have been deposited to the ProteomeXchange Consortium *via* the PRIDE partner repository with the dataset identifier PXD004515.
(TIFF)
