# Supplementary figures and images for: Is Transthyretin a Regulator of Ubc9 SUMOylation?
Source: PLoS One. 2016 Aug 8;11(8):e0160536. doi: 10.1371/journal.pone.0160536 (PMC4976990; doi:10.1371/journal.pone.0160536)

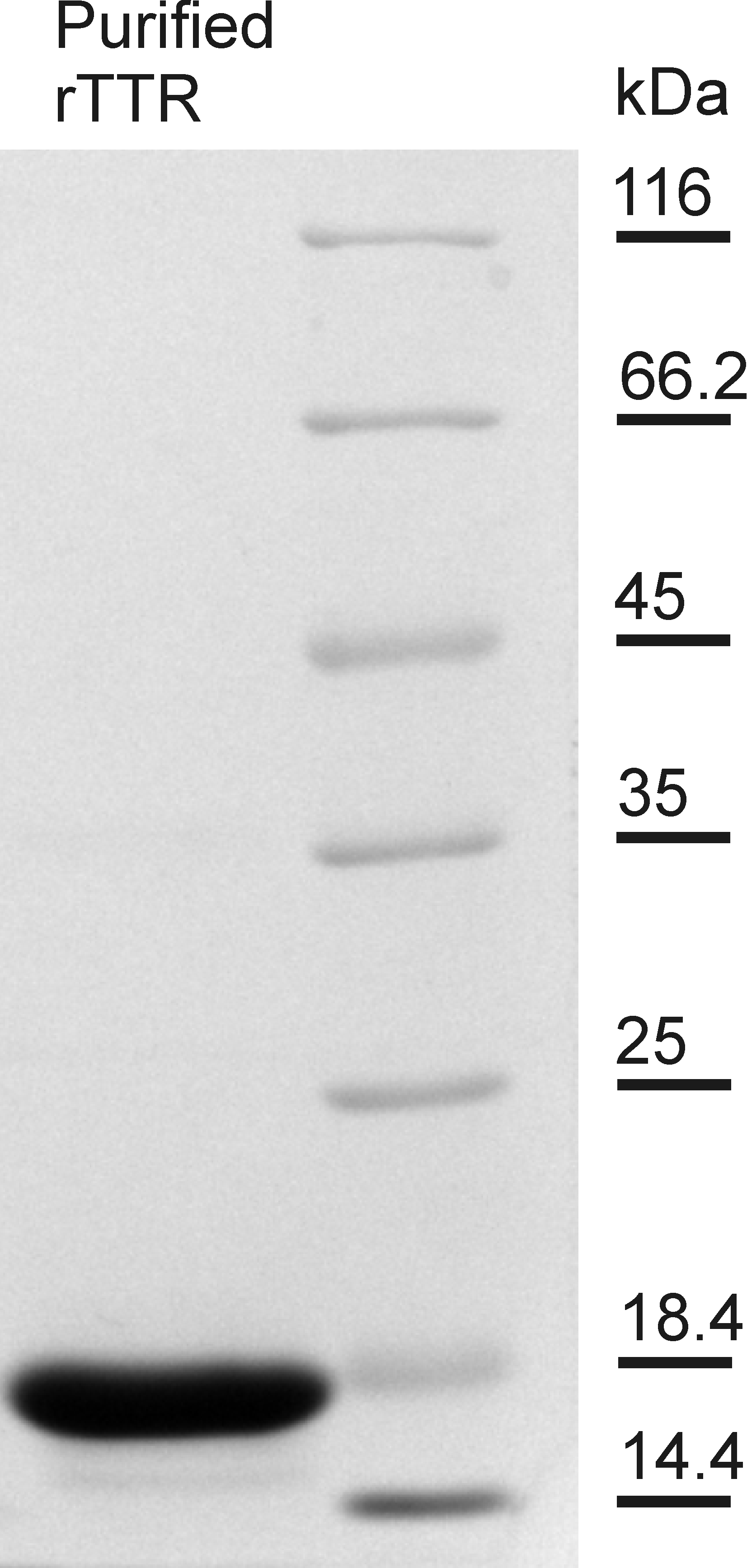

Supplement: S1 Fig — The sample containing 10 μg of recombinant TTR possessing a C-terminal histidine tag (rTTR), purified as described in the Materials and Methods, was heated in Laemmli gel loading buffer for 30 min at 95°C and loaded on 12% SDS gel. After electrophoresis the gel was stained with Coomassie blue R250. (TIF) [file pone.0160536.s001.TIF]

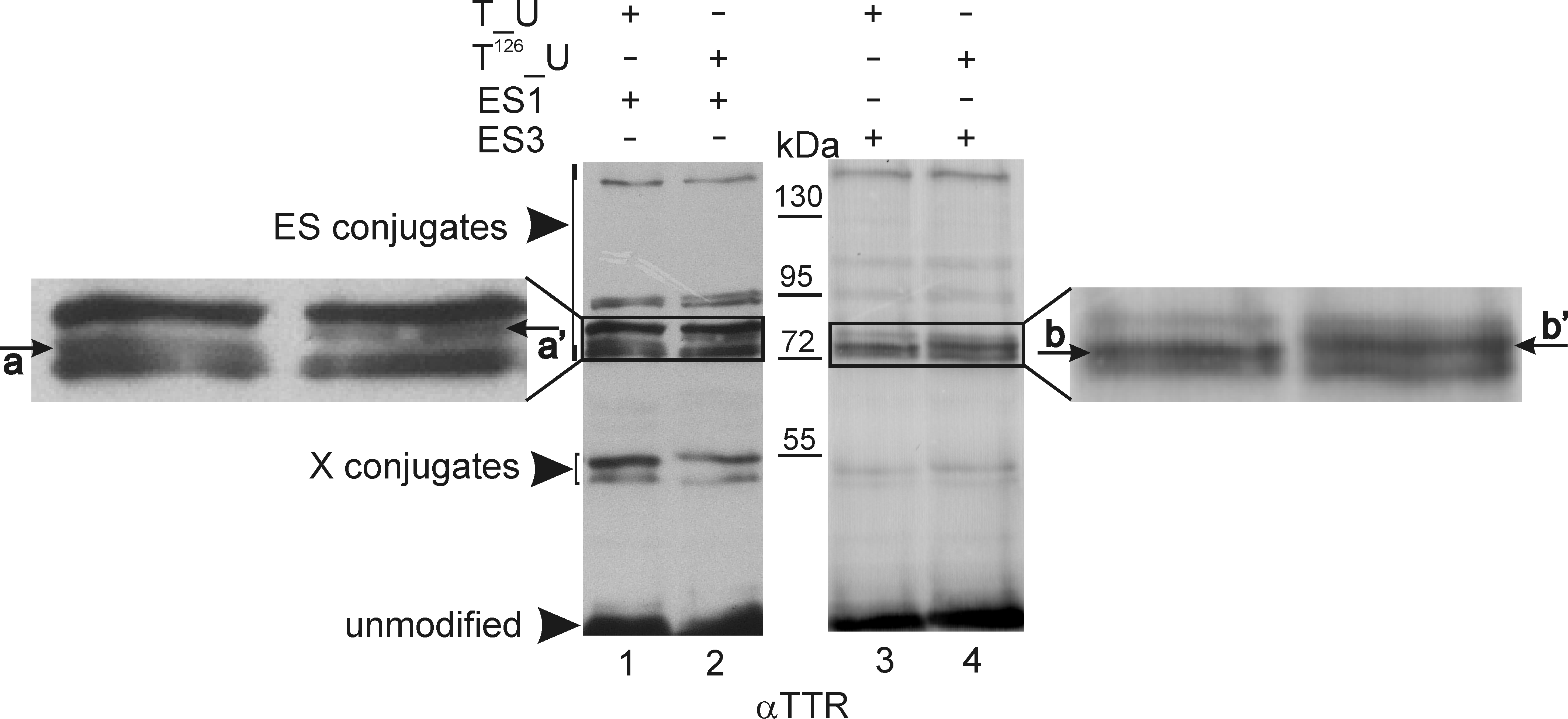

Supplement: S2 Fig — Non-mutated TTR_Ubc9 (T_U) or the K126R mutant of TTR in TTR126_Ubc9 (T126_U) were co-expressed in HEK293 cells with EGFP-labelled SUMO-1 (ES1) or SUMO-3 (ES3). The cell lysates were analysed by WB using anti-TTR antibodies (αTTR). The outlined areas have been enlarged. The letters indicate changes in the mobility of the bands resulting from the K126R mutation in TTR. ES conjugates and X conjugates indicate forms of the fusion proteins modified by EGFP-labelled SUMO or endogenous modifiers, respectively. (TIF) [file pone.0160536.s002.TIF]

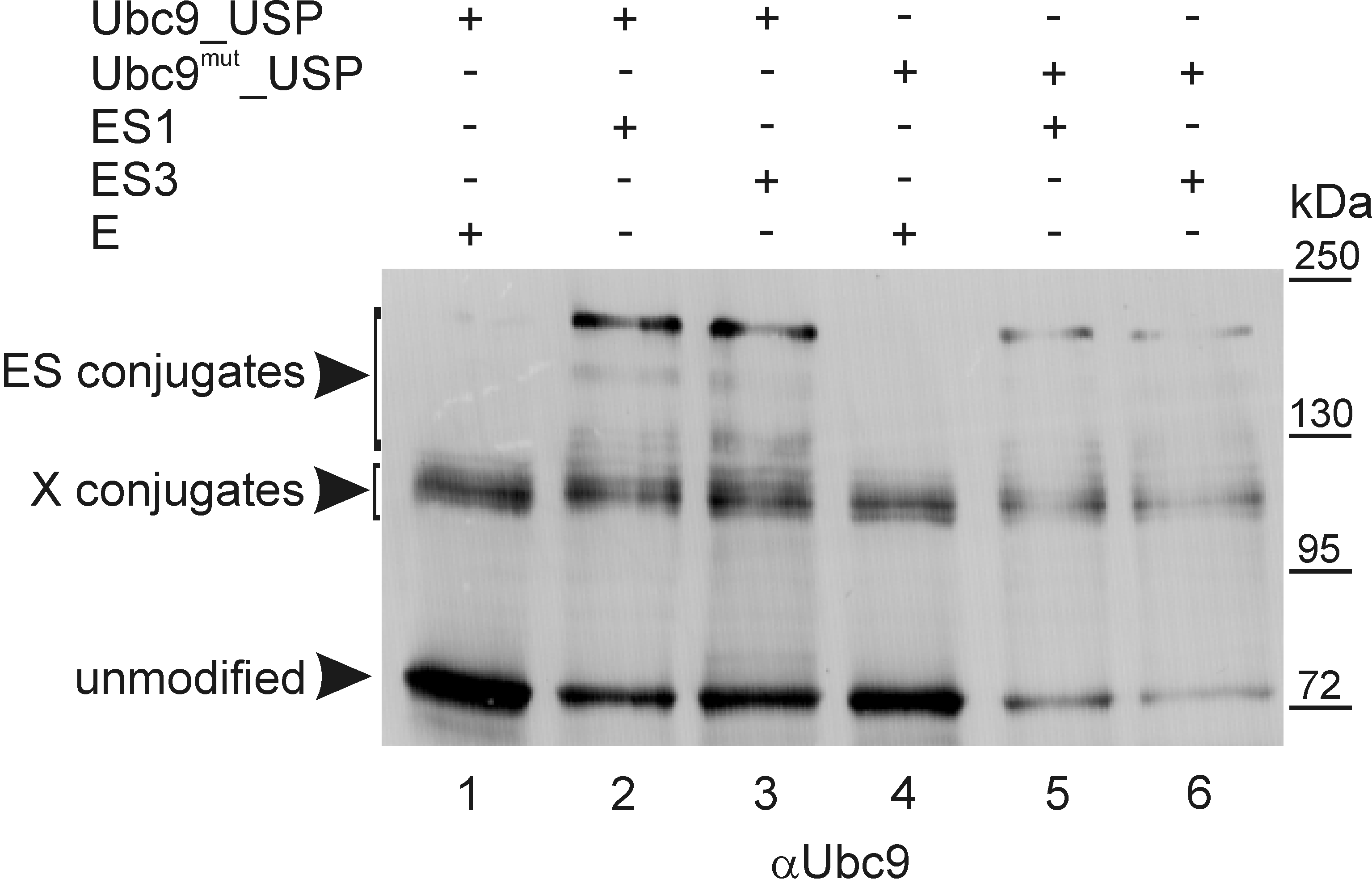

Supplement: S3 Fig — Drosophila melanogaster Ultraspiracle protein (USP) fused to non-mutated (Ubc9) or to the K14,153,154R mutant of Ubc9 (Ubc9mut) located at the C-terminus (Ubc9_USP and Ubc9mut_USP) was co-expressed with EGFP-labelled SUMO-1 (ES1), SUMO-3 (ES3) or EGFP (E) in the HEK293 cell line. Cell lysates were analysed by WB using anti-Ubc9 antibodies (αUbc9). ES conjugates and X conjugates indicate proteins modified by EGFP-labelled SUMO or endogenous modifiers, respectively. (TIF) [file pone.0160536.s003.TIF]

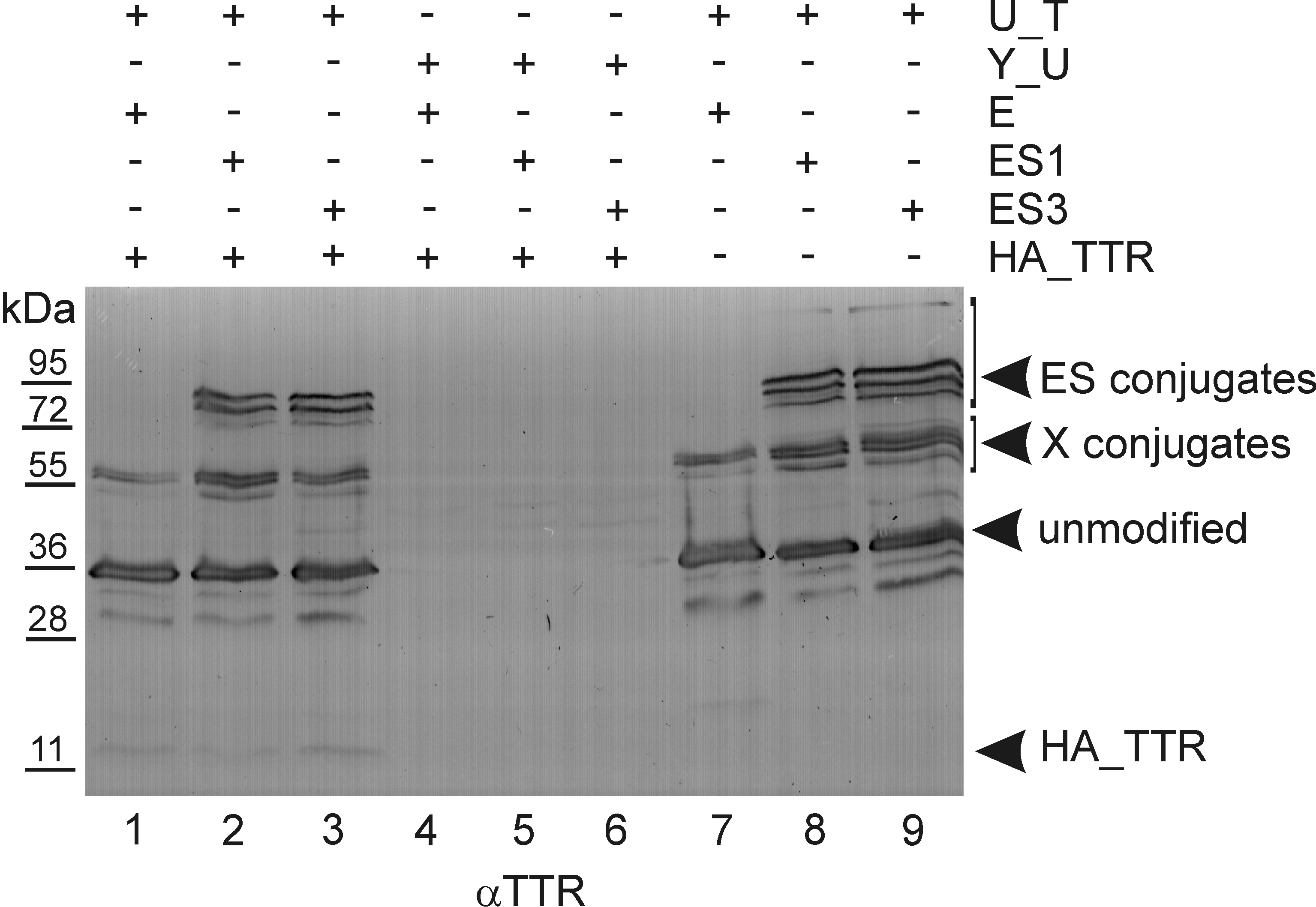

Supplement: S4 Fig — Non-fused HA-labelled TTR (HA_TTR) was co-expressed with EGFP-labelled SUMO-1 (ES1), SUMO-3 (ES3), EGFP (E) or EYFP-labelled Ubc9 (Y_U) in the absence or presence of TTR fused to the C-terminus of Ubc9 (U_T). The cell lysates were analysed by WB using anti-TTR antibodies (αTTR). ES conjugates and X conjugates indicate forms of the fusion proteins modified by EGFP-labelled SUMO or endogenous modifiers, respectively. (TIF) [file pone.0160536.s004.TIF]

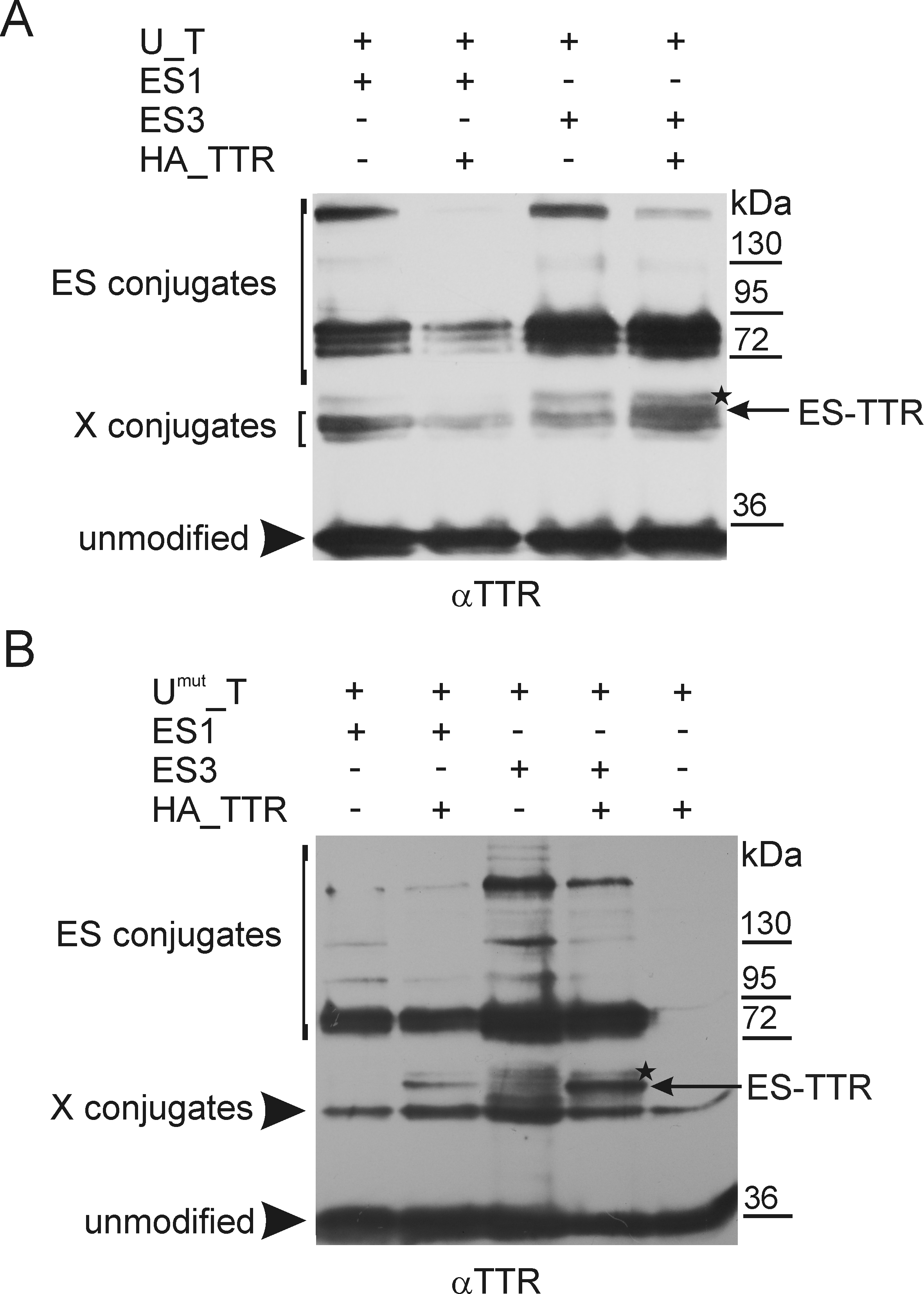

Supplement: S5 Fig — Exposure on photographic film of (A) the WB presented in Fig 6A and (B) WB performed using equivalent samples of the same transfection experiment as presented in Fig 6B. TTR fused to the C-terminus of non-mutated (U_T) (A) or the K14,153,154R mutant of Ubc9 (Umut_T) (B) was co-expressed with EGFP-labelled SUMO-1 (ES1) or SUMO-3 (ES3) in the absence or presence of non-fused HA-labelled TTR (HA_TTR). The cell lysates were analysed by WB using anti-TTR antibodies (αTTR). The asterisks indicate truncated SUMOylated proteins. ES conjugates and X conjugates represent proteins modified by EGFP-labelled SUMO or endogenous modifiers, respectively. (TIF) [file pone.0160536.s005.TIF]

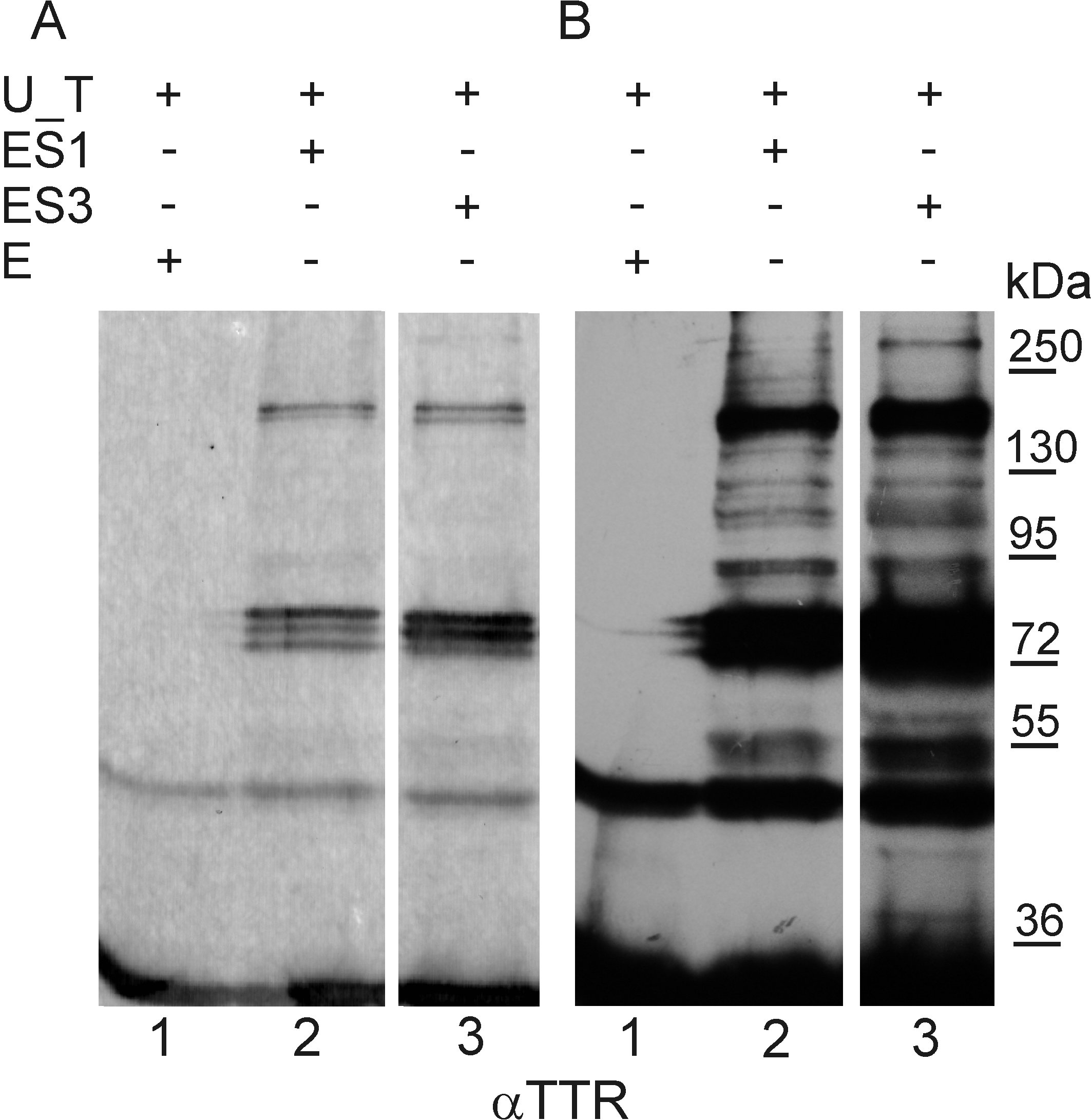

Supplement: S6 Fig — TTR fused to the C-terminus of Ubc9 (U_T) was co-expressed in HEK293 cells with EGFP-labelled SUMO-1 (ES1), SUMO-3 (ES3) or EGFP (E). Cell lysates were analysed by WB using anti-TTR antibodies (αTTR). (A) Fluorescence scans and (B) exposure on photographic film. (TIF) [file pone.0160536.s006.TIF]

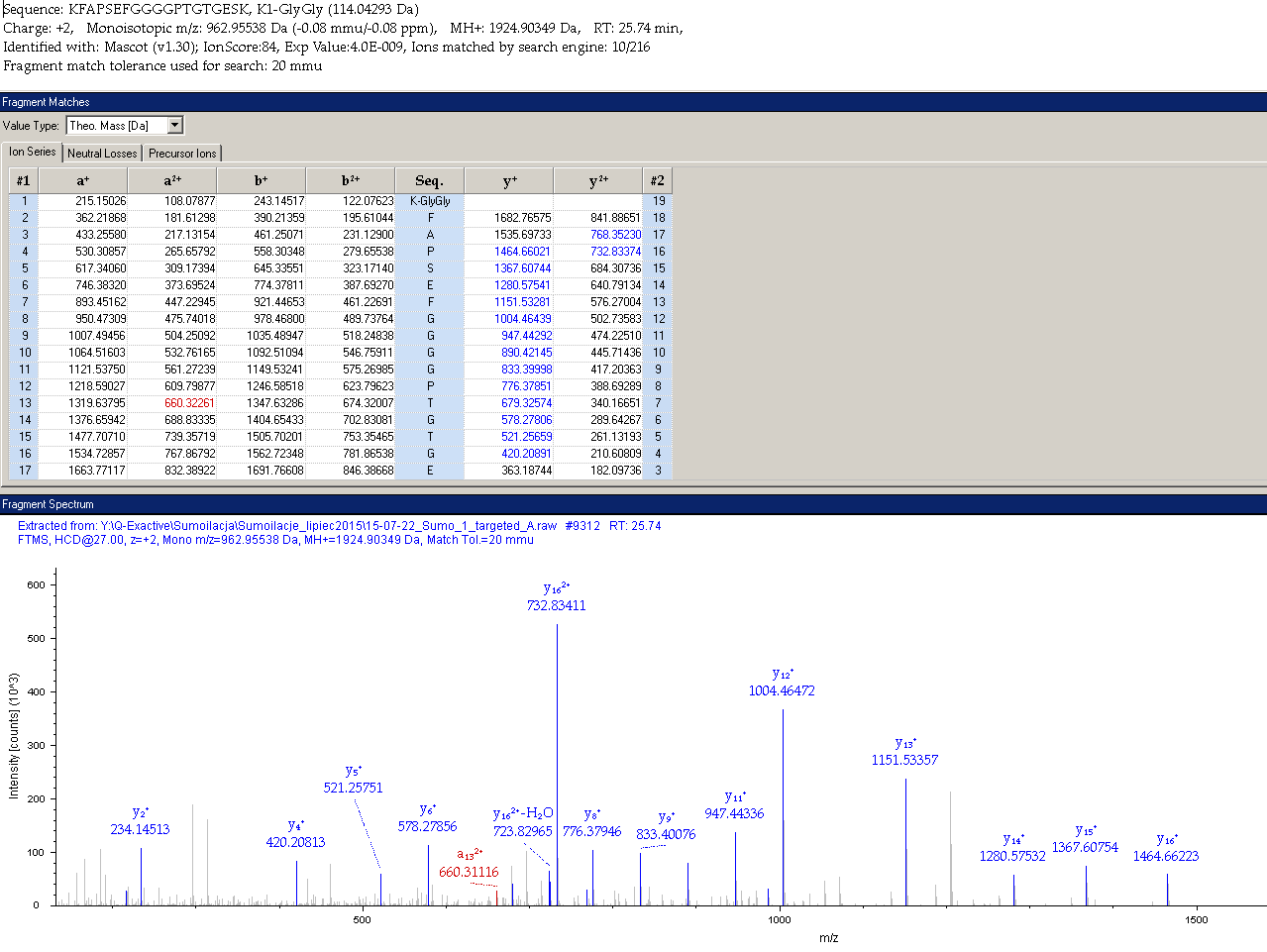

Supplement: S7 Fig — Annotated MS/MS spectra of the precursor ion at m/z 962.955 (2+) correspond to peptide k(GG)FAPSEFGGGGPTGTGESK. The peptide was assigned to the MS/MS spectrum with a MASCOT ion score of 84. The mass spectrometry proteomics data have been deposited to the ProteomeXchange Consortium via the PRIDE partner repository with the dataset identifier PXD004515. (TIFF) [file pone.0160536.s007.tiff]
